# Woodland strawberry WRKY71 acts as a promoter of flowering via a transcriptional regulatory cascade

**DOI:** 10.1038/s41438-020-00355-4

**Published:** 2020-09-01

**Authors:** Yingying Lei, Yiping Sun, Baotian Wang, Shuang Yu, Hongyan Dai, He Li, Zhihong Zhang, Junxiang Zhang

**Affiliations:** Liaoning Key Laboratory of Strawberry Breeding and Cultivation, College of Horticulture, Shenyang Agricultural University, 120 Dongling Road, Shenyang, 110866 China

**Keywords:** Flowering, Plant sciences

## Abstract

The WRKY proteins are a large family of transcription factors that play important roles in stress responses and plant development. However, the roles of most WRKYs in strawberry are not well known. In this study, *FvWRKY71* was isolated from the woodland strawberry ‘Ruegen’. *FvWRKY71* was highly expressed in the shoot apex and red fruit. Subcellular localization analysis showed that FvWRKY71 was located in the nucleus. Transactivation analysis showed that FvWRKY71 presented transcriptional activation activity in yeast. Overexpression of *FvWRKY71* in Arabidopsis and woodland strawberry revealed early flowering in the transgenic plants compared with the wild-type control. Gene expression analysis indicated that the transcript levels of the flowering time and development integrator genes *AP1*, *LFY*, *FT*, *AGL42*, *FUL*, *FPF1*, *SEP1*, *SEP2*, and *SEP3* were increased in *FvWRKY71-*overexpressing Arabidopsis and strawberry plants compared with the wild-type controls, which may result in accelerated flowering in transgenic plants. Furthermore, FvWRKY71 was proven to directly bind to the W-boxes (TTGACT/C) of the *FvFUL*, *FvSEP1*, *FvAGL42*, *FvLFY*, and *FvFPF1* promoters in vitro and in vivo. Taken together, our results reveal a transcriptional regulatory cascade of FvWRKY71 involved in promoting flowering in woodland strawberry.

## Introduction

The WRKY proteins are a family of plant transcription factors (TFs). Members of the family harbor one or two conserved WRKY domains, which contain a conserved WRKYGQK heptapeptide sequence at the N-terminus and a C_2_H_2_ or C_2_HC zinc-finger protein motif at the C-terminus^1^. Based on the number of WRKY domains and the structure of the C-terminal zinc-finger motif, the WRKY family is divided into three categories: groups I, II, and III. Group II proteins are further classified as IIa, IIb, IIc, IId, or IIe proteins based on the primary amino acid sequence^2^. It has been more than 20 years since the identification of the first WRKY protein^1^. Since that time, diverse biological functions of WRKY proteins in different species have been studied^3^.

WRKY proteins play crucial roles in plant signaling, the regulation of plant development and the responses to various biological and abiotic stresses^4^. In Arabidopsis, most *WRKY* genes have been shown to be involved in defense against pathogens^2^. For example, *AtWRKY33* can modulate host defenses against *Alternaria brassicicola* and *Botrytis cinerea* infection^5,6^. AtWRKY46 forms a complex with AtWRKY70 and AtWRKY53 to positively regulate basal resistance to *Pseudomonas syringae*^7^. *AtWRKY8*, *AtWRKY25*, *AtWRKY33*, and *AtWRKY63* are involved in the response to abiotic stress^8–11^. *AtWRKY6*, *AtWRKY44*, *AtWRKY57*, and *AtWRKY71* are involved in plant developmental processes^2,12–14^. In rice, at least five *OsWRKY* genes have been demonstrated to participate in the defense response against pathogens^15,16^. In addition, many *OsWRKY* genes are regulators of the response to abiotic stresses, such as heat, drought, and salt^16–18^. Most WRKY genes are involved in the stress response, but some participate in the determination of flowering time. In Arabidopsis, WRKY75 positively regulates flowering via the GA-mediated signaling pathway^19^. AtWRKY12 is a positive factor in the regulation of flowering, whereas AtWRKY13 is a negative factor in the modulation of flowering under short-day conditions. Further analysis indicated that both WRKY12 and WRKY13 can directly bind to the promoter of *FUL* and produce different effects on their downstream target genes^20^. The heterologous overexpression of *WRKY12* in *Miscanthus lutarioriparius* in Arabidopsis promotes flowering. Consistent with the early flowering phenotype, the expression levels of the *APETALA 1 (AP1)*, *FLOWERING LOCUS T (FT)*, *LEAFY (LFY)*, and *FRUITFULL (FUL)* genes are significantly increased in transgenic Arabidopsis plants^21^. AtWRKY71 accelerates flowering by regulating the flowering genes *FT* and *LFY*^22^. The overexpression of *CpWRKY71* in Arabidopsis also causes an early flowering, precocious leaf senescence phenotype^23^. The potential pathways of the WRKY TFs involved in flowering are not fully understood.

The molecular mechanism of flowering has been extensively studied in the annual model plant Arabidopsis, whereas less is known about the molecular control of flowering in perennial species, such as strawberry. Strawberry plants can be divided into two main groups based on their flowering habits: seasonal flowering (SF) and perpetual flowering (PF) strawberry. SF strawberry flowers under short days and is induced to flower in autumn^24^. SF patterns exist in diploid woodland strawberry (*Fragaria vesca*) and cultivated strawberry (*Fragaria* × *ananassa*), which also exhibit genomes with a high degree of colinearity^25–27^. In *F. vesca*, SF is caused by a single repressor gene: *SEASONAL FLOWERING LOCUS* (*SFL*)^28^. *TERMINAL FLOWER1* (*TFL1*), a candidate gene for *SFL*, is activated to inhibit strawberry flowering in summer and is suppressed to induce flower initiation in autumn^29^. The knockdown of *FvSOC1* produces a continuous flowering phenotype, which is similar to mutant of the floral repressor *FvTFL1*^30^. In SF strawberry, *FvFT1* and *FaFT1* are homologous genes of *FT* in Arabidopsis. PF strawberry produces new inflorescences continuously throughout the growing season from spring until late autumn. In the diploid *Fragaria vesca*, the PF trait is caused by a recessive mutation in the *SFL* gene^31^. A major locus controlling PF in cultivated strawberry is the perpetual flowering and runnering (*PFRU*) locus, which modulates the balance between sexual and asexual plant reproduction^27,31^. *FaPFRU* shows opposite effects on flowering and runnering, exerting a positive effect on flowering and a negative effect on runnering, indicating that the two traits are genetically linked and share common physiological control^31^.

To date, the only available information on the WRKY family in woodland strawberry has come from the genome-wide analysis of the expression of WRKY genes in different developmental stages or under biotic and abiotic stresses^32,33^ and the potential roles of the WRKY genes in woodland strawberry are largely unknown. In the annual model plant Arabidopsis, *WRKY71* can promote flowering. However, whether *WRKY71* presents a similar function or regulatory mechanism in flowering in perennial plants, such as strawberries, is still largely unknown. In addition, the expression of *FvWRKY46*, another name of *FvWRKY71*, is upregulated during fruit development and ripening based on RNA-seq and RT-qPCR results^32^, which indicates that *FvWRKY71* may play a role in regulating fruit development and ripening, in addition to promoting flowering. To investigate the role of *WRKY71* in *F. vesca*, the *FvWRKY71* gene was overexpressed in Arabidopsis and woodland strawberry, and we found that *FvWRKY71* conferred early flowering. We also found that FvWRKY71 accelerated flowering through the direct activation of different flowering-related genes between strawberry and Arabidopsis. These data will enrich the regulatory network of WRKY71 in different plants and provide some information on the promotion of flowering in Rosaceae plants.

## Results

### Structural analysis of FvWRKY71

To investigate the function of the FvWRKY71 protein, we first identified and cloned *FvWRKY71* from the woodland strawberry ‘Ruegen’ by RT-PCR using the specific primers shown in Supplemental Table S1. The coding sequence of *FvWRKY71* was 1107 bp in length, which encoded a protein of 368 amino acids. The estimated molecular weight and isoelectric point were 41.62 and 6.46 kDa, respectively.

**Fig. 1:**
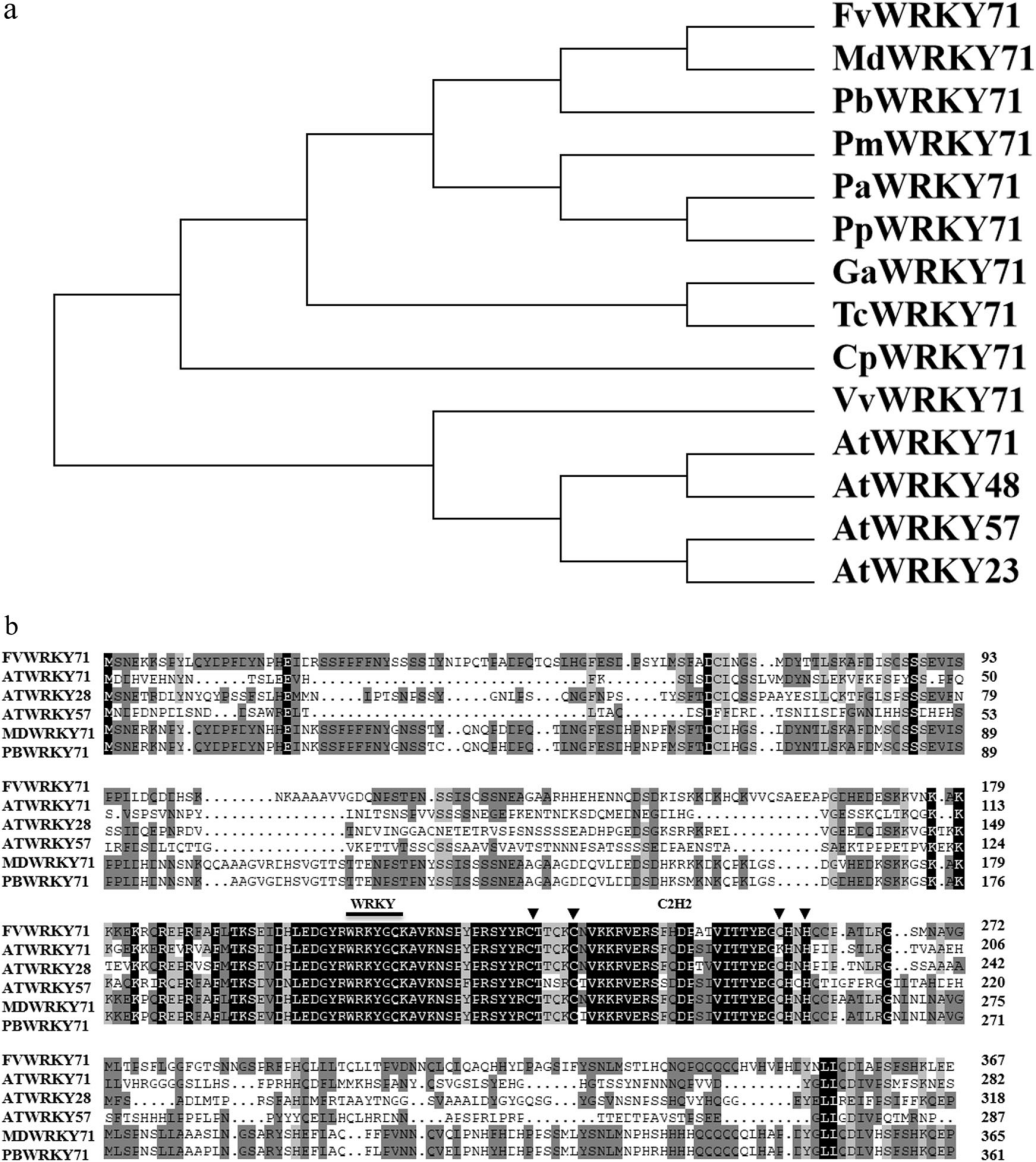
Phylogenetic and structural analyses of FvWRKY71. **a** Phylogenetic tree based on the alignment of amino acid sequences among WRKY proteins. **b** Multiple alignment of WRKY proteins from higher plants

To examine the evolutionary relationship between FvWRKY71 and its orthologs from multiple species, we performed multiple-sequence alignment using their full-length amino acid sequences. A phylogenetic tree was constructed through neighbor-joining analysis (Fig. 1a). The phylogenetic tree results indicated that FvWRKY71 exhibited the closest genetic relationship to the WRKY71 proteins from apple and pear, which also belong to the Rosaceae family (Fig. 1a). FvWRKY71 was also homologous to Arabidopsis WRKY71, WRKY48, WRKY57, and WRKY23 according to BLAST analysis in the TAIR database.

To better understand the conserved domain of FvWRKY71, we used DNAMAN software to carry out amino acid sequence analysis. FvWRKY71 was shown to contain a conserved WRKYGQ domain and a conserved C_2_H_2_-type zinc-finger motif (Fig. 1b). Therefore, FvWRKY71 identified in this study contains one WRKY domain and belongs to group II^34^ (Fig. 1a).

### Expression patterns of FvWRKY71 in strawberry

**Fig. 2:**
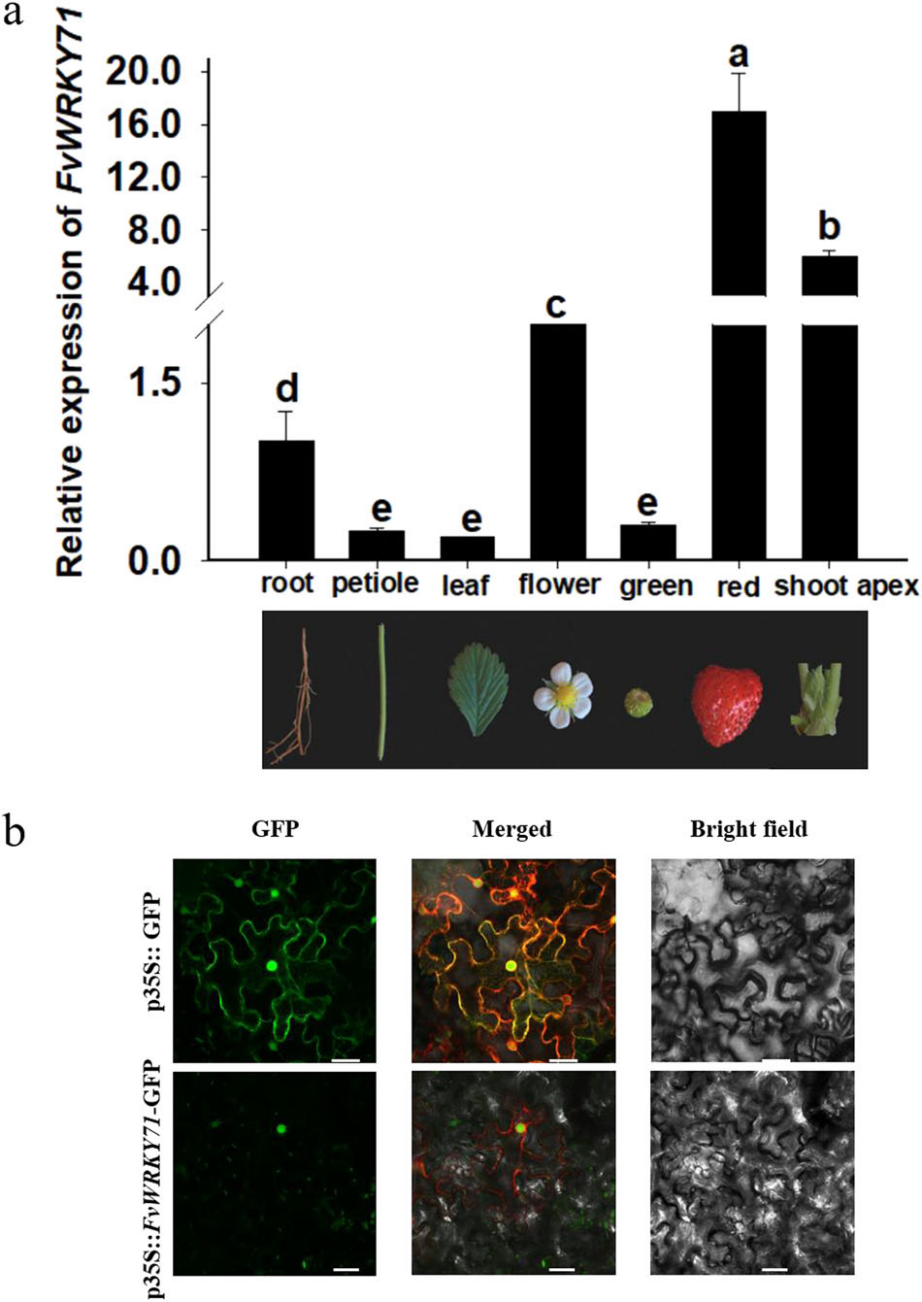
Expression pattern and subcellular localization of FvWRKY71. **a** Expression pattern of *FvWRKY71* in different organs. RT-qPCR analysis was used to test the relative expression level of *FvWRKY71* in various organs. Values are the mean ± SD from three independent experiments with three biological replicates. **b** Subcellular localization of the FvWRKY71 protein. p35S::GFP was transformed into tobacco (top). p35S::FvWRKY71-GFP was transformed into tobacco (bottom), and the fusion protein was located in the nucleus. Bar = 30 μm

Different developmental stages and different organs were selected to detect the expression patterns of the *FvWRKY71* gene by RT-qPCR (Fig. 2a). The expression levels in the flowers, shoot apex, and red fruits were approximately 2-, 6- and 17-fold higher than that in the roots, respectively. Lower expression levels were found in the roots, petioles, leaves, and green fruits. (Fig. 2a).

### Subcellular localization of FvWRKY71

To examine the subcellular localization of the FvWRKY71 protein, we transiently expressed the recombinant FvWRKY71-GFP protein in *Nicotiana benthamiana* leaves. The fusion protein signal was examined by confocal microscopy. As shown in Fig. 2b, the FvWRKY71-GFP fusion protein only existed in the nucleus, while the green signal of the GFP control was distributed in the nucleus and the cell membrane. The results indicate that FvWRKY71 localizes to the nucleus (Fig. 2b).

### Transcriptional activation activity of FvWRKY71 in yeast

**Fig. 3:**
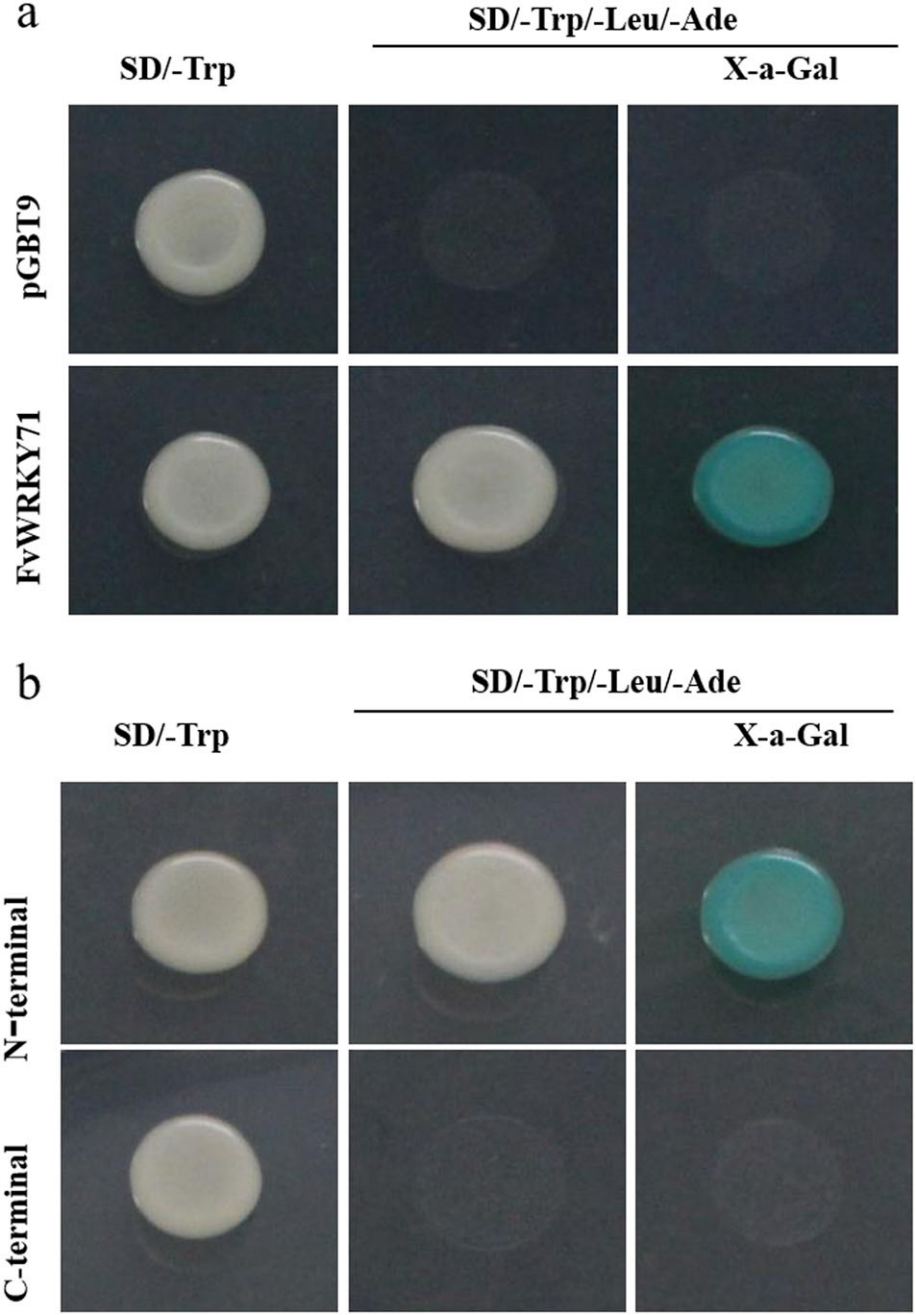
Transactivation activation activation analysis of FvWRKY71. The construct of pBD-FvWRKY71 was transformed into yeast Y2H Gold cells, and the cells were then examined on SD/-Trp and SD/-Trp/-Leu/-Ade/X-α-gal plates. **a** Transactivation activation analysis of FvWRKY71. **b** Transactivation activation analysis of the N-terminus and C-terminus of FvWRKY71

A yeast system was used to test whether FvWRKY71 exhibited transcriptional activation activity. The complete coding region of *FvWRKY71* was fused to the GAL4 DNA-binding domain in the pGBT9 vector, and the construct was then transformed into Y2H Gold cells. The empty pGBT9 vector served as a control. As shown in Fig. 3a, yeast cells transformed with the pBD-FvWRKY71 fusion vector grew well on both SD/-Trp and SD/-Trp/-Leu/-Ade media. They also exhibited blue colonies on alpha-galactosidase plates (Fig. 3a), which indicated that they presented alpha-galactosidase activity. In contrast, the control yeast strain was only viable on SD/-Trp medium. These data showed that FvWRKY71 exhibited transcriptional activation activity in yeast. To further elucidate which segments presented transcriptional activation activity, we transformed the N-terminus and C-terminus of *FvWRKY71* in the pGBT9 vector into yeast cells. The results showed that the N-terminus showed transcriptional activation activity, while the C-terminus did not (Fig. 3b).

### Overexpression of *FvWRKY71* in Arabidopsis promotes flowering

**Fig. 4:**
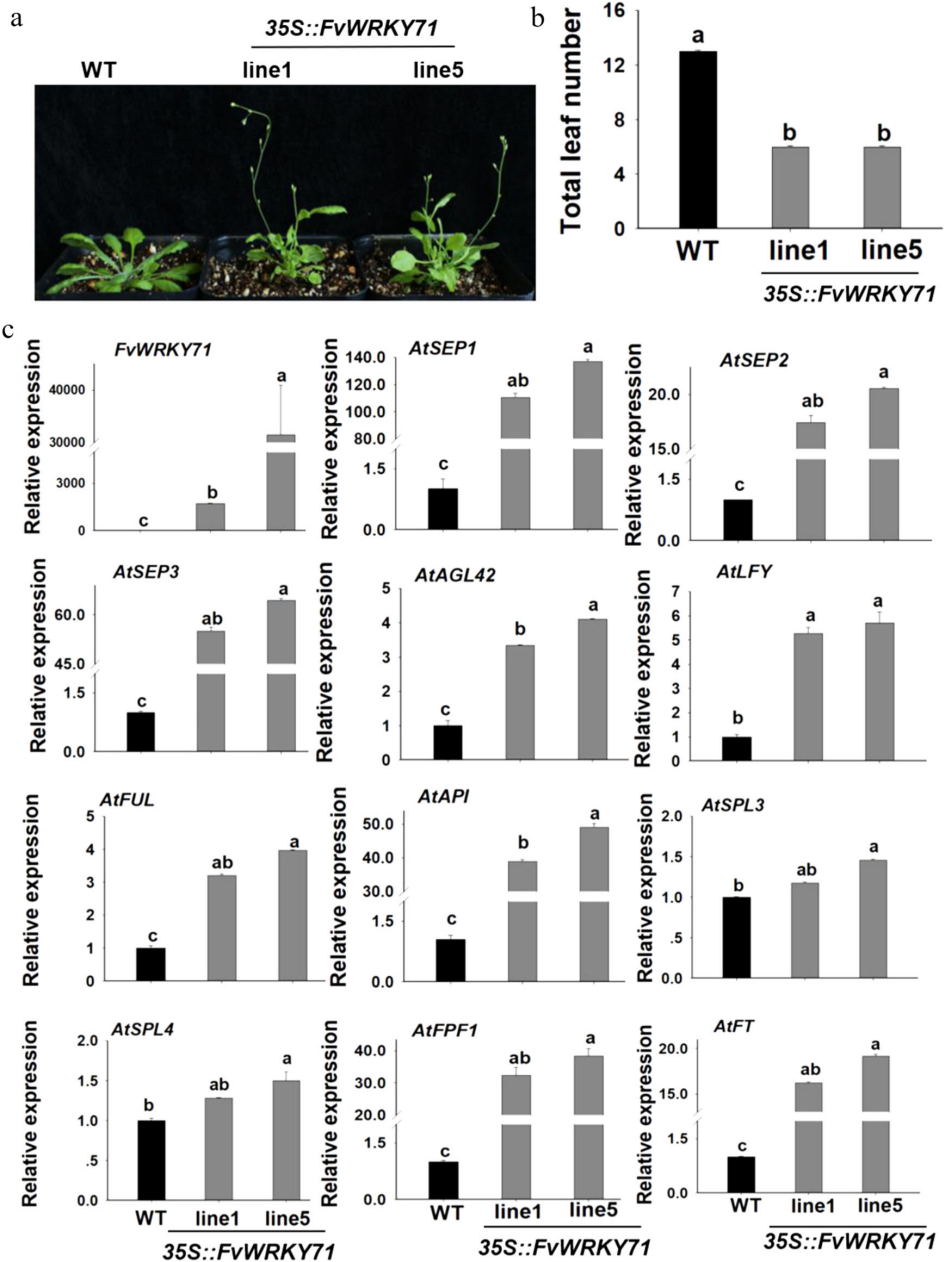
The phenotypes of Arabidopsis *FvWRKY71-*overexpressing plants and gene expression analysis. **a** The phenotypes of Arabidopsis *FvWRKY71-*overexpressing transgenic lines and wild-type control plants. **b** The number of rosette leaves at flowering in transgenic plants and wild-type plants. **c** RT-qPCR analysis of *FvWRKY71* and genes involved in flowering, including *AtSEP1*, *AtSEP2*, *AtSEP3*, *AtAGL42*, *AtAP1*, *AtLFY*, *AtFUL*, *AtSPL3*, *AtSPL4*, *AtFPF1*, and *AtFT*, between wild-type and transgenic Arabidopsis plants. Values are the mean ± SD from three independent experiments with three biological replicates. Different letters indicate significant differences (*P* < 0.05, based on Duncan’s multiple range test)

To investigate the biological function of the *FvWRKY71* gene, we constructed a p35S::FvWRKY71 overexpression vector (Fig. S1a) and transformed it into Arabidopsis to obtain transgenic plants. The transgenic Arabidopsis lines were examined by PCR to determine on DNA levels (Fig. S1b) and by RT-qPCR to determine on RNA levels (Fig. 4c). Two T_3_ homozygous positive transgenic lines were selected for further phenotypic analysis. By observing transgenic lines 1 and 5, we found that the ectopic expression of the *FvWRKY71* gene in Arabidopsis induced an early flowering phenotype compared with wild-type plants (Fig. 4a). Under long (16 h) daylight conditions, *FvWRKY71-*overexpressing transgenic plants started bolting at 19 days after sowing, whereas wild-type plants started bolting at 33 days after sowing. In addition, the number of rosette leaves at flowering in the transgenic lines was approximately seven, while the number of rosette leaves at flowering in the wild-type plants was approximately thirteen (Fig. 4b). These data indicate that the overexpression of *FvWRKY71* in Arabidopsis can promote flowering.

To investigate whether the overexpression of *FvWRKY71* in Arabidopsis affected the transcript levels of flowering-related genes for the early flowering phenotype. We searched the Arabidopsis flowering database (http://www.phytosystems.ulg.ac.be/florid/) for candidate genes responsible for accelerating flowering in *FvWRKY71*-overexpressing transgenic lines. Twelve genes involved in the determination of flowering time and flower development were identified by RT-qPCR analysis. The expression levels of *SEPALLATA1 (SEP1)*, *SEPALLATA2 (SEP2)*, *SEPALLATA3 (SEP3)*, *AGMOUS-LIKE 42 (AGL42)*, *AP1*, *LFY*, *FUL*, *FLOWERING PROMOTING FACTOR 1 (FPF1)*, and *FT* were shown to be significantly increased compared with those in wild-type plants (Fig. 4c). The expression level of *FvWRKY71* in transgenic line 5 was higher than that in transgenic line 1, and the expression of downstream genes was also higher in transgenic line 5 than in transgenic line 1 (Fig. 4c).

### Overexpression of *FvWRKY71* in woodland strawberry accelerates flowering

**Fig. 5:**
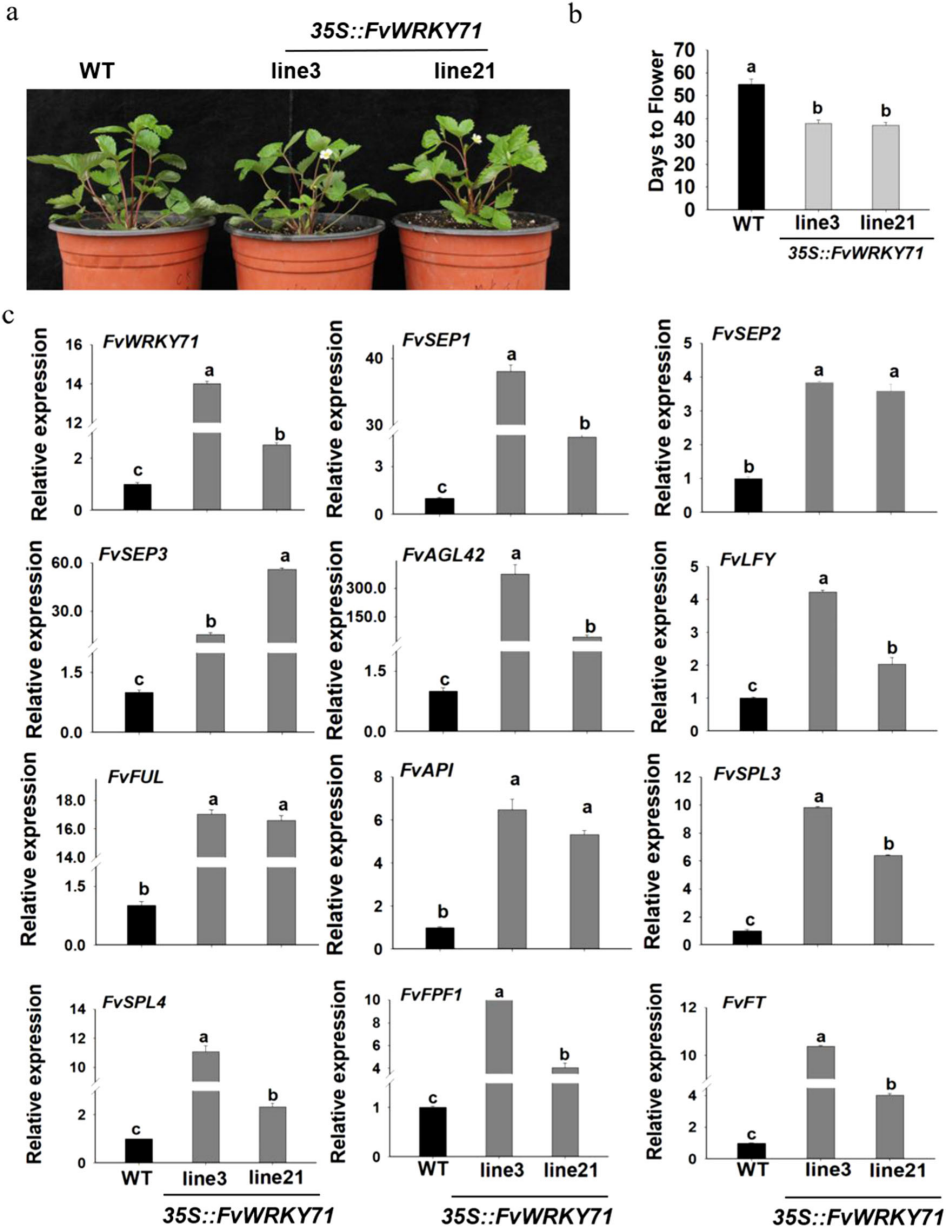
The phenotypes of *FvWRKY71-*overexpressing transgenic woodland strawberry lines and gene expression analysis. **a** The phenotypes of *FvWRKY71-*overexpressing woodland strawberry lines and wild-type plants. **b** Days to flowering between woodland strawberry transgenic lines and wild-type plants after transfer from culture medium. **c** RT-qPCR analysis of *FvWRKY71* and genes involved in flowering between wild-type and transgenic woodland strawberry plants, including *FvSEP1*, *FvSEP2*, *FvSEP3*, *FvAGL42*, *FvAP1*, *FvLFY*, *FvFUL*, *FvSPL3*, *FvSPL4*, *FvFPF1,* and *FvFT*. Values are the mean ± SD from three independent experiments with three biological replicates. Different letters indicate significant differences (*P* < 0.05, based on Duncan’s multiple range test)

To further investigate the function of *FvWRKY71*, we also overexpressed *FvWRKY71* in ‘Ruegen’ strawberry. The transgenic lines were first examined by PCR to detect DNA levels (Fig. S1c). Next, we investigated transgenic lines 3 and 21 by RT-qPCR (Fig. 5c). The transcript level of *FvWRKY71* was significantly increased in transgenic lines 3 and 21 compared with the non-transgenic control plants (Fig. 5c). Interestingly, we observed that the *FvWRKY71*-overexpressing transgenic lines promoted flowering in woodland strawberry relative to wild-type plants (Fig. 5a), and the early flowering phenotype was consistent with the results of *FvWRKY71* overexpression in Arabidopsis. We found that transgenic line 3, transgenic line 21, and wild-type plants took 38, 37, and 55 days, respectively, to start flowering under the same conditions (Fig. 5b). Next, we tested the expression levels of *SEP1*, *SEP2*, *SEP3*, *AGL42*, *AP1*, *LFY*, *FUL*, *SQUAMOSA PROMOTER BINDING PROTEIN-LIKE3 (SPL3)*, *SPL4*, *FPF1*, and *FT* in *FvWRKY71-*overexpressing strawberry transgenic lines by RT-qPCR. We found that the transcript abundance of these flowering-related genes was significantly increased in transgenic strawberry lines compared with wild-type control plants (Fig. 5c), which was in agreement with the results of the overexpression of *FvWRKY71* in Arabidopsis (Fig. 5c). We also analyzed the expression of *FvSOC1* and *FvTFL1* in transgenic strawberry plants by RT-qPCR. We found that the expression of *FvSOC1* and *FvTFL1* was significantly reduced in transgenic line 3 compared with that in wild-type plants, whereas there was no considerable difference between transgenic line 21 and wild-type plants (Fig. S3). Taken together, these data reveal that *FvWRKY71* promotes flowering and regulates the expression of flowering-related genes.

### *FvWRKY71* accelerates the flowering of woodland strawberry mainly via the direct activation of *FvFUL*, *FvSEP1*, *FvAGL42*, *FvLFY*, and *FvFPF1*

**Fig. 6:**
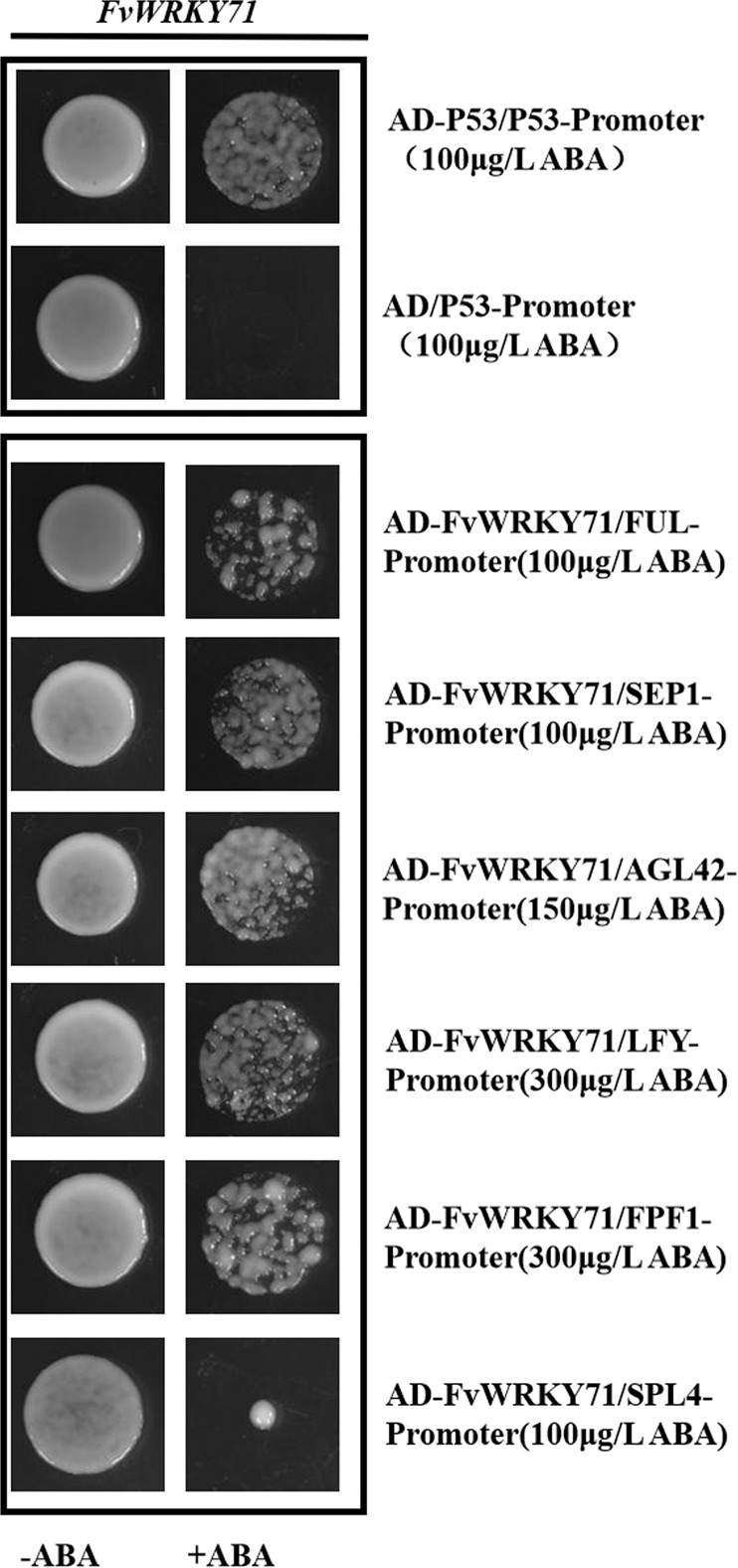
Yeast one-hybrid assay to test whether FvWRKY71 could directly bind to the promoters of *FvFUL*, *FvSEP1*, *FvAGL42*, *FvLFY*, *FvFPF1*, and *FvSPL4*. pAD-FvWRKY71 was cotransformed into yeast Y1H strains in combination with proFvFUL-pAbAi, proFvSEP1-pAbAi, proFvAGL42-pAbAi, proFvLFY-pAbAi, proFvFPF1-pAbAi, or proFvSPL4-pAbAi. The transformed strains all grew on SD/-Leu selective medium containing aureobasidin A (ABA) (100, 100, 150, 300, 300, and 100 μg/L, respectively)

To further identify the genes directly regulated by FvWRKY71 for early flowering in woodland strawberry, we investigated the promoter sequences of *FvSEP1*, *FvSEP2*, *FvSEP3*, *FvAGL42*, *FvAP1*, *FvLFY*, *FvFUL*, *FvSPL3*, *FvSPL4*, *FvFPF1*, and *FvFT* to determine whether they contained W-boxes (TTGACT/C). We found that *FvFUL*, *FvSEP1*, *FvAGL42*, *FvLFY*, *FvFPF1*, and *FvSPL4* exhibit W-boxes in these promoter regions (Supplemental File S1). A yeast one-hybrid experiment was first conducted to detect whether FvWRKY71 could bind to the promoters of *FvFUL*, *FvSEP1*, *FvAGL42*, *FvLFY*, *FvFPF1*, and *FvSPL4*. pAD-FvWRKY71 was cotransformed into yeast Y1H strains in combination with proFvFUL-pAbAi, proFvSEP1-pAbAi, proFvAGL42-pAbAi, proFvLFY-pAbAi, proFvFPF1-pAbAi, or proFvSPL4-pAbAi. The first five transformed strains were able to grow on SD/-Leu selective medium containing aureobasidin A (ABA) (100, 100, 150, 300, and 300 μg/L, respectively), whereas that transformed with *FvSPL4* (100 μg/L ABA) could not (Fig. 6), which indicates that FvWRKY71 can bind to the promoters of *FvFUL*, *FvSEP1*, *FvAGL42*, *FvLFY*, and *FvFPF1*.

**Fig. 7:**
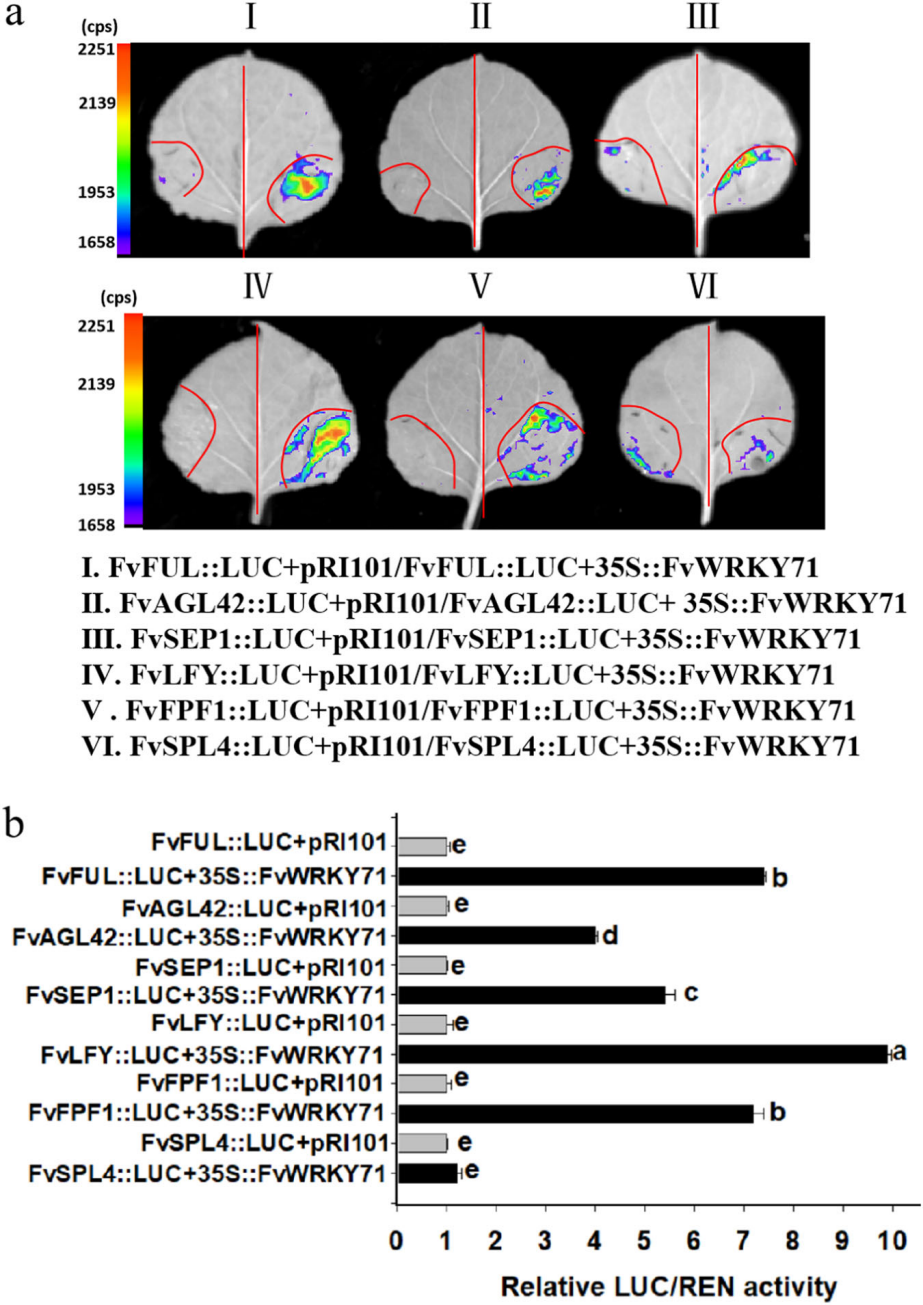
Luciferase activity assay for determining whether FvWRKY71 could directly bind to the promoters of *FvFUL*, *FvSEP1*, *FvAGL42*, *FvLFY*, *FvFPF1*, and *FvSPL4*. **a** Tobacco transient expression assay. p35S::FvWRKY71 was cotransformed into *N. benthamiana* in combination with proFvFUL::LUC, proFvAGL42::LUC, proFvSEP1::LUC, proFvLFY::LUC, proFvFPF1::LUC, or proFvSPL4::LUC. **b** The comparison of luciferase activity. The transcriptional activity of these infiltrated tobacco leaves based on the ratio of LUC to REN was investigated with a dual luciferase reporter gene assay kit. Different letters indicate significant differences (*P* < 0.05, based on Duncan’s multiple range test)

A luciferase assay is an important method for detecting the binding of transcription factors and specific domain sites in the promoters of target genes^35^. To clarify the effect of FvWRKY71 on the promoters of *FvFUL*, *FvSEP1*, *FvAGL42*, *FvLFY*, *FvFPF1*, and *FvSPL4*, we performed luciferase reporter assays in *N. benthamiana* leaf cells. We constructed proFvFUL-LUC, proFvSEP1-LUC, proFvAGL42-LUC, proFvLFY-LUC, proFvFPF1-LUC and proFvSPL4-LUC reporters and the effector plasmid 35S:FvWRKY71 (Fig. S1). As expected, FvWRKY71 exerted activation effects on *FvFUL*, *FvSEP1*, *FvAGL42*, *FvLFY*, and *FvFPF1* in these LUC reporter assays, but not on *FvSPL4* (Fig. 7a), and FvWRKY71 activated the expression of the *FvFUL*, *FvAGL42*, *FvSEP1*, *FvLFY*, and *FvFPF1* genes to various levels (7.4-, 4.0-, 5.4-, 9.9-, and 7.2-fold, respectively) compared with the corresponding control (Fig. 7b). Together, these data suggest that FvWRKY71 accelerates the flowering of woodland strawberry mainly through the direct activation of *FvFUL*, *FvAGL42*, *FvSEP1*, *FvLFY*, and *FvFPF1*.

## Discussion

Flowering is a key step in plant growth and development. Early flowering is an important trait for Rosaceae crops such as strawberry, apple, and peach. Here, we identified the transcription factor WRKY71 in woodland strawberry and found that *FvWRKY71* was expressed in all examined organs, especially in the shoot apex and red fruit. In Arabidopsis, the transcript level of *AtWRKY71* gradually increased with seedling development from 4 to 16 days and reached the highest level at 16 days, which is consistent with the floral transition^22^. The higher expression of *FvWRKY71* in the shoot apex is consistent with the floral transition. Functional analysis by overexpressing *FvWRKY71* in Arabidopsis and strawberry revealed that *FvWRKY71* plays an important role in accelerating flowering.

Flower induction is crucial for plant growth and development, and can promote the shift from vegetative growth to reproductive growth^36,37^. Flower induction is regulated by many exogenous and endogenous factors. In the model plant Arabidopsis, at least five flowering pathways have been found, including the photoperiod, gibberellin, vernalization, autonomous, and age pathways^38–40^. These pathways are related to various signals, such as photoperiod, age, temperature, abiotic stress and hormones, and regulate the expression of factors involved in flowering. These flowering pathways can be regulated independently from each other or in an interrelated manner, thus forming a complex flowering regulation network. The functional analysis of genes representing various flowering pathways did not reveal large differences between diploid woodland strawberry and cultivated strawberry^25,26,41^.

In perennial plants, molecular studies on flowering have focused on the *FT*, *LFY*, *TFL1*, and *SOC1* genes. As a key component of flowering pathways, the expression of *FT* is influenced by many factors, some of which act as positive regulators and others as repressors^42–47^. The signal is passed on to *FvAP1* and *FvFUL* to effect the transition from the vegetative to the reproductive stage^29,48^. The *FT* gene is highly expressed in the leaves in cultivated strawberry and is induced by long days and high temperatures^26^. Decreasing or increasing *LFY* transcript abundance can delay or accelerate flowering, respectively^49^. It has been reported that Arabidopsis WRKY75 can promote flowering via *FT* and *SOC1* but not *LFY*. The promoter of *FT* contains some W-box cis-elements, and *FT* is the direct target of AtWRKY75. In addition, AtWRKY75 partially controls GA signaling in the regulation of flowering time^19^. Arabidopsis WRKY12 and WRKY13 modulate flowering in opposite directions under short-day conditions. AtWRKY12 and AtWRKY13 can interact with two DELLA proteins, AtGAI and AtRGL1, to affect the transcriptional activity of *AtWRKY12* and *AtWRKY13*, which regulate the expression of *AtFUL*, a direct downstream target gene of *AtWRKY12* and *AtWRKY13*, to control flowering^20^. The transcription levels of *AP1* and *LFY* are higher in WRKY71-1D than in wild-type plants, and AtWRKY71 promotes flowering through the direct activation of *FT* and *LFY*^22^. In our study, the relative expression levels of *FT*, *LFY*, and *FUL* in the Arabidopsis and strawberry transgenic lines were also higher than those in the wild-type (Figs. 4c and 5c). We identified W-boxes in the *LFY* promoter region, while we could not find a W-box in the *FT* promoter in woodland strawberry. There are three W-boxes in the Arabidopsis *FT* promoter region^22^. Therefore, FvWRKY71 could not directly regulate *FT* gene expression by binding to W-box elements in woodland strawberry and might affect the transcript level of *FT* in other unidentified ways. The overexpression of *FUL* in Arabidopsis can promote flowering^50,51^. Interestingly, we found that the strawberry *FUL* gene presented two W-boxes in its promoter region (Supplemental File S1) and showed that FvWRKY71 could interact with the *FvFUL* promoter to modulate *FvFUL* expression. These findings indicate that different WRKY proteins regulate the transcript levels of different floral meristem identity genes or floral integrators to control their distinct flowering pathways and that some WRKY proteins may be involved in controlling the timing of GA-mediated flowering.

*TFL1* and *SOC1* function as major repressors of flowering in perennial species, including strawberry and rose^52–54^. We found that the expression levels of *FvTFL1* and *FvSOC1* were significantly reduced in woodland strawberry transgenic line 3 compared with wild-type plants, which is consistent with the early flowering phenotype of *FvWRKY71*-overexpressing plants. However, there were no large differences between transgenic line 21 and wild-type plants (Fig. S3). The higher expression of *FvWRKY71* in transgenic line 3 (14.0-fold higher than wild-type control) than transgenic line 21 (2.5-fold higher than wild-type control) may result in lower expression of *FvTFL1* and *FvSOC1* in transgenic line 3. In Arabidopsis, *SUPPRESSOR OF OVEREXPRESSION OF CONSTANS1* (*SOC1*) mediates both endogenous and exogenous signals to accelerate flowering^30^. These findings reveal that the functions of important flowering genes may not be completely the same between annual and perennial plants.

During the transition to flowering, *FPF1* regulates flowering through an independent pathway that is parallel to that of *LFY* and *AP1*^55^. We found W-boxes in the promoter sequences of the *FPF1* gene in woodland strawberry, and we showed that FvWRKY71 can directly regulate *FvFPF1* expression by binding to the W-boxes of the *FvFPF1* promoter. The SPL3 and SPL4 transcription factors can also directly bind to the promoter of the *LFY* gene^55^. SPL3 directly activates *LFY*, *FUL*, and *AP1* expression in Arabidopsis^56^. The expression levels of *SPL3* and *SPL4* are significantly increased in *FvWRKY71*-overexpressing lines, and a W-box exists in the *FvSPL4* promoter. However, FvWRKY71 cannot bind to the W-box of the *FvSPL4* gene (Figs. 6 and 7). In Arabidopsis, the promoter region of the *AP1* gene contains five W-boxes, whereas AtWRKY71 cannot bind to any of the AP1 W-boxes^22^. These findings indicate that the *FvSPL4* and *AtAP1* genes contain W-box elements but that they are not targets of WRKY71 in woodland strawberry and Arabidopsis. WRKY71 might regulate the expression of these genes to accelerate flowering in indirect ways.

MADS domain transcription factors play important roles in flower development, the control of flowering time and fruit development^57^. In our study, we observed that many MADS-domain proteins, such as *AP1*, *AGL42*, *SEP1*, *SEP2*, and *SEP3*, exhibited different expression levels between *FvWRKY71-*overexpressing transgenic plants and wild-type plants (Figs. 4c and 5c). It has been reported that the MADS-domain proteins AP1, AP3, PI, AG, and SEP3 play important roles in floral tissues to modulate flower development^58^. SEP1, SEP2, and SEP3 encode MADS box transcription factors, which are a group of organ identity genes that are required for flower development. LFY directly activates *SEP1*, *SEP2*, and *SEP3* in Arabidopsis^59^. AGL42 encodes MADS-box transcription factors and is closely related to *SOC1* in Arabidopsis^60^. Here, we found that the upstream regulatory region of woodland strawberry *AP1* does not contain a W-box, while there are five W-boxes in the Arabidopsis *AP1* promoter. Additionally, we found that the promoter regions of *FvAGL42* and *FvSEP1* contain W-boxes (Supplemental File S1) and that FvWRKY71 is able to directly control the expression of *FvAGL42* and *FvSEP1* by binding to W-boxes in the promoters of *FvAGL42* and *FvSEP1* (Figs. 6 and 7). Therefore, these results indicate that FvWRKY71 promotes flowering through the direct activation of different flowering-related genes between strawberry and Arabidopsis.

**Fig. 8:**
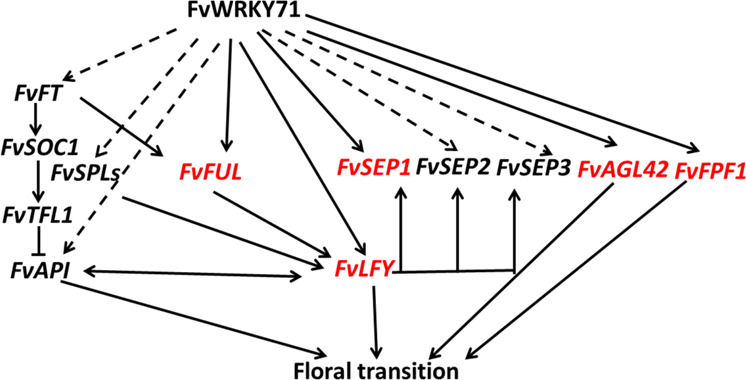
Proposed transcriptional regulatory cascade whereby FvWRKY71 regulates flowering in woodland strawberry. FvWRKY71 accelerates flowering via the direct activation of *FvFUL*, *FvLFY*, *FvFPF1*, *FvAGL42*, and *FvSEP1* (red font) and the indirect regulation of *FvFT*, *FvSPL3*, *FvSPL4*, *FvAP1*, *FvSEP2*, and *FvSEP3* (dotted line). The other solid lines indicate regulatory relationships reported in the literature^29,48,51,52,55,59,60^. Arrows represent activation, and bars represent repression

Together, our results show that FvWRKY71 acts as a positive regulator of flowering in woodland strawberry. The *FUL*, *SEP1*, *AGL42*, *LFY*, and *FPF1* gene homologs of the flowering pathway genes of Arabidopsis were analyzed in strawberry. We found that these genes were highly expressed in *FvWRKY71*-overexpressing transgenic plants. The transcriptional regulatory cascade of FvWRKY71 involved in accelerating flowering in woodland strawberry (Fig. 8) differs from the regulatory mechanism of WRKY71 in promoting flowering in Arabidopsis. FvWRKY71 promotes flowering via the direct modulation of *FvFUL*, *FvSEP1*, *FvAGL42*, *FvLFY*, and *FvFPF1* expression. These data will enrich the known regulatory network of WRKY71 in different plants and provide information on the promotion of flowering on Rosaceae plants.

## Materials and methods

### Plant materials and growth conditions

The material used in this study was the diploid woodland strawberry (*Fragaria vesca*) ‘Ruegen’. The strawberries were grown in the greenhouse at Shenyang Agricultural University, China. *FvWRKY71*-overexpressing transgenic strawberry plants and wild-type plants were transferred from culture medium to the soil substrate and cultivated in an incubator at 23 °C (16 h/8 h, light/dark). Thirty days later, the plants were transplanted to the greenhouse of Shenyang Agricultural University in June. The days to flowering were recorded in the transgenic plants (six for line 3 and six for line 21) and six wild-type plants in the greenhouse after transfer from culture medium.

Wild-type (WT) Arabidopsis (Columbia) seeds were synchronized at 4 °C for 2 days and then placed at 22 °C ± 2 °C under long-day (16 h/8 h, light/dark) conditions.

The tobacco species used for transient expression analysis was *Nicotiana benthamiana*, which was grown at 23 °C under a long-day photoperiod (16 h/8 h, light/dark) for 1 month.

### Phylogenetic analysis and multiple-sequence alignment

The full-length sequences of WRKY71 proteins from different plants were aligned by using ClustalW (http://www.clustal.org/), and the phylogenetic tree was constructed using MEGA6.0 software (http://www.megasoftware.net/)^61^. The neighbor-joining (NJ) method was used to produce a phylogenetic tree with the following parameters: bootstrap analysis (1,000 replicates; random seeds), Poisson correction and pairwise deletion^61^. The FvWRKY71 orthologs used for phylogenetic tree construction came from *Arabidopsis thaliana*, *Prunus persica*, *Malus x domestica*, *Pyrus bretschneideri*, *Prunus mume*, *Prunus avium*, *Vitis vinifera*, *Theobroma cacao*, *Gossypium arboreum*, and *Carica papaya*. DNAMAN software was used to carry out amino acid sequence alignments between FvWRKY71 and different species (version 6.0; Lynnon Biosoft, Quebec, Canada).

### Subcellular localization

The coding region without the stop codon of *FvWRKY71* was amplified by RT-PCR from the red fruit of ‘Ruegen’ based on woodland strawberry genome information^62,63^. The FvWRKY71-F and FvWRKY71-R primers are shown in Supplemental Table S1. The products were inserted into the pRI101-GFP vector with *EcoR*I and *Kpn*I to generate the plasmid p35S::FvWRKY71-GFP. The pRI101-GFP and p35S::FvWRKY71-GFP plasmids were transformed into *Agrobacterium tumefaciens* strain EHA105. The leaves of 1-month-old tobacco (*N. benthamiana*) plants were infiltrated with *A. tumefaciens* cells harboring pRI101-GFP and pRI101-FvWRKY71-GFP. The GFP fluorescence signal was observed by confocal fluorescence microscopy (Leica DMi8 A, Wetzlar, Germany) 2 days after transient transformation.

### Transactivation assay

The coding region of *FvWRKY71* was amplified by RT-PCR from the red fruit of ‘Ruegen’ using specific primers (Supplemental Table S1) and then inserted into the pGBT9 vector. The pGBT9-FvWRKY71 plasmid and the pGBT9 vector were introduced into Y2H Gold cells using the PEG/LiAc method^64^, and the cells were grown on SD/-Trp agar plates. The pGBT9 vector was used as a negative control. After 3 days, single transformed yeast colonies were diluted with 10 μL sterile water, and a 3-μL aliquots were dropped onto SD/-Trp and SD/-Trp/-Leu/-Ade/X-a-Gal plates to observe yeast growth at 30 °C for 3–4 days. Next, we amplified the coding sequences of the N-terminus and C-terminus of *FvWRKY71* by RT-PCR from the red fruit of ‘Ruegen’ (Supplemental Table S1), and the PCR products were cloned into the pGBT9 vector to identify the activation area at the N-terminus or C-terminus.

### Overexpression vector construction and plant transformation

The full-length coding region of *FvWRKY71* was amplified by RT-PCR from the red fruit of ‘Ruegen’ and cloned into pRI101-AN to generate the overexpression plasmid p35S::FvWRKY71. The PCR primer sets are shown in Supplemental Table S1. The placsmid was confirmed by sequencing. The plasmid was introduced into *A. tumefaciens* strain GV3101 via the freeze-thaw method and transformed into Arabidopsis via the floral dip method^65^. Positive transgenic plants were screened on plates containing 1/2 MS medium with 30 mg/L kanamycin. Furthermore, two T_3_ generation transgenic Arabidopsis lines (Line 1 and Line 5) were detected by RT-qPCR for gene expression analysis. The numbers of rosette leaves were analyzed in each plant after the first inflorescence emergence. Ten plants of each line were used for analyzing the numbers of rosette leaves.

To further elucidate the function of *FvWRKY71*, we introduced the overexpression plasmid p35S::FvWRKY71 into ‘Ruegen’ strawberry as described previously^66^. Explants were placed on medium supplemented with 250 mg/L timetin and 250 mg/L cefotaxime and 10 mg/L kanamycin for selection culture in the dark. When adventitious buds appeared, they were placed under light. The concentration of kanamycin was increased from 10 to 30 mg/L in subsequent subcultures. After ~8 months of selective culture, we obtained transgenic plants and transferred them to the greenhouse, where they were grown under short-day conditions. The positive transgenic strawberry plants were evaluated by PCR to detect DNA levels and RT-qPCR for gene expression analysis.

### RNA isolation and expression analysis

To test the expression of representative genes involved in flowering, young leaves of the transgenic plants and wild-type plants were chosen. Total RNA was isolated from three mixed samples using the modified CTAB method as described previously^67^. Each mixed sample was obtained from six transgenic plants or wild-type plants. Total RNA (1 μg) was reverse-transcribed in a 20-μL reaction using a PrimeScript^TM^ RT reagent Kit (Takara, Japan). The 10-μL reaction for removing genomic DNA contained 2.0 μL of 5× gDNA Eraser Buffer, 1.0 μL gDNA Eraser, 1 μg RNA, and RNase-free ddH_2_O to 10 μL. The 20-μL reverse transcription reaction contained 10 μL of the previous reaction product, 1.0 μL PrimeScript RT Enzyme Mix1, 1.0 μL RT Primer Mix, 4.0 μL of 5× PrimeScript Buffer 2, and 4.0 μL of RNase-free ddH_2_O. The following thermal profile was used for reverse transcription: 42 °C for 5 min, followed by 37 °C for 30 min and 85 °C for 5 s. RT-qPCR was performed in an ABI 7500 system (Applied Biosystems, Foster City, CA, USA). All cDNA samples were diluted with ddH_2_O (V:V; 1:4). RT-qPCR was performed in a volume of 10 μL containing 5 μL UltraSYBR Green Mixture reagent (ComWin Biotech, Beijing), 0.5 μL cDNA, 1 μL of the primer set, and 3.5 μL ddH_2_O, and relative expression levels were calculated using the 2^–ΔΔCt^ method, taking the WT plants as a control. Each sample was quantified in triplicate with three biological replicates. The reaction conditions for RT-qPCR consisted of an initial step (50 °C for 2 min, 95 °C for 10 min), followed by 40 cycles (95 °C for 15 s, 60 °C for 1 min) and a denaturing step (95 °C for 15 s, 60 °C for 15 s, 95 °C for 15 s). We performed normalization first with Arabidopsis 18S rRNA as a control and then with strawberry 26S rRNA as a control^68^. The gene primers used for the RT-qPCR experiments are listed in Supplemental Table S1.

### Yeast one-hybrid assay

The coding sequence of *FvWRKY71* was obtained through RT-PCR with specific primers from the red fruit of ‘Ruegen’. Then, the fragment was digested with *EcoR*I and *BamH*I and inserted into the pGAD424 vector to obtain pAD-FvWRKY71. The promoter regions of *FvFUL*, *FvSEP1*, *FvAGL42*, *FvLFY*, *FvFPF1*, and *FvSPL4* were amplified by PCR from the genomic DNA of the leaves of ‘Ruegen’, verified by sequencing and cloned into the pAbAi vector with the *EcoR*I and *BamH*I restriction enzymes. The primers used for the yeast one-hybrid analysis are listed in Supplemental Table S1.

The plasmids proFUL-pAbAi, proSEP1-pAbAi, proAGL42-pAbAi, proLFY-pAbAi, proFPF1-pAbAi, and proSPL4-pAbAi were introduced into the Y1H Gold yeast strain using the PEG/LiAC method^61^ and grown on SD/-Ura agar plates. After 3 days, we transferred positive yeast to agar plates containing SD/-Ura/+AbA (50–500 μg/L) and conducted screening to determine the minimum inhibitory concentration of AbA in relation to yeast growth. Next, we transformed pAD-FvWRKY71 into yeast strains containing proFUL-pAbAi, proSEP1-pAbAi, proAGL42-pAbAi, proLFY-pAbAi, proFPF1-pAbAi, and proSPL4-pAbAi and plated them in SD/-Leu/and SD/-Leu/+AbA plates. Finally, we observed the growth of the yeast on agar plates. The Yeast Protocols Handbook (Clontech) was used to perform the above yeast experiments.

### Dual-luciferase reporter system

Two kilobase sequences of the *FvFUL*, *FvSEP1*, *FvAGL42*, *FvLFY*, *FvFPF1*, and *FvSPL4* promoters containing W-boxes (TTGACT/C) were amplified from the genomic DNA of the leaves of ‘Ruegen’. The fragments were digested with *Hind*ΙΙΙ and *BamH*I and inserted into the pGreenII0800-LUC vector as reporter plasmids^35^. The overexpression vector p35S::FvWRKY71 was used as an effector plasmid.

These plasmids were introduced into *A*. *tumefacien*s strain GV3101 via the freeze-thaw method. *Agrobacterium* strain GV3101 carrying the effector plasmid (empty vector or p35S::FvWRKY71) and the specific reporter plasmid (ProFvFUL::LUC, ProFvSEP1::LUC, ProFvAGL42::LUC, ProFvLFY::LUC, ProFvFPF1::LUC, and ProFvSPL4::LUC) was cultured to OD_600_ = 1.0 at 28 °C. The cells were harvested and resuspended in medium (1 M MgCl_2_, 100 mM acetosyringone and 1 M MES, pH 5.6). Then, the solution was placed at room temperature without shaking for 2 h and infiltrated into 1-month-old *N. benthamiana* leaves. The infiltrated tobacco plant was placed in the dark for 24 h and then placed under light for 48 h. We tested luciferase signaling with a living fluorescence imager (Lb985, Berthold, Germany). The transcriptional activity in the infiltrated tobacco leaves indicated by the ratio of LUC to REN was investigated with a dual luciferase reporter gene assay kit (Beyotime, China). Six biological repeats were measured for each sample. The primers used for the LUC/REN activity analysis are listed in Supplemental Table S1.

### Accession numbers

AT5G62165 (AGL42), AT1G69120 (AP1), AT5G24860 (FPF1), AT1G65480 (FT), AT5G60910 (FUL), AT5G61850 (LFY), AT5G15800.2 (SEP1), AT3G02310 (SEP2), AT1G24260 (SEP3), AT2G33810 (SPL3), AT1G53160 (SPL4), FvH4_7g28740.1 (AGL42), FvH4_4g29600.1 (AP1), FvH4_2g30770.1 (FPF1), FvH4_6g00090.1 (FT), FvH4_5g13500.1 (FUL), FvH4_5g09660.1 (LFY), FvH4_7g28690.1 (SEP1), FvH4_6g46420.1 (SEP2), FvH4_4g23530.1 (SEP3), FvH4_3g08880 (SPL3), FvH4_3g19650.1 (SPL4), and FvH4_7g12700.1 (SOC1). GenBank: JN172097.1 (FvTFL1),

## Supplementary information

Supplementary Information
