# Supplementary material for: Woodland strawberry WRKY71 acts as a promoter of flowering via a transcriptional regulatory cascade
Source: Hortic Res. 2020 Sep 1;7:137. doi: 10.1038/s41438-020-00355-4 (PMC7458929; doi:10.1038/s41438-020-00355-4)
Supplement: Supplementary file 1 — Supplementary Information [file 41438_2020_355_MOESM1_ESM.docx]

**Supplemental Fig. S1.** Identification of the overexpression of FvWRKY71 in Arabidopsis and strawberry **a** Overexpression vector construction. **b** PCR test of the overexpression of *FvWRKY71* in Arabidopsis. **c** PCR test of the overexpression of *FvWRKY71* in strawberry.


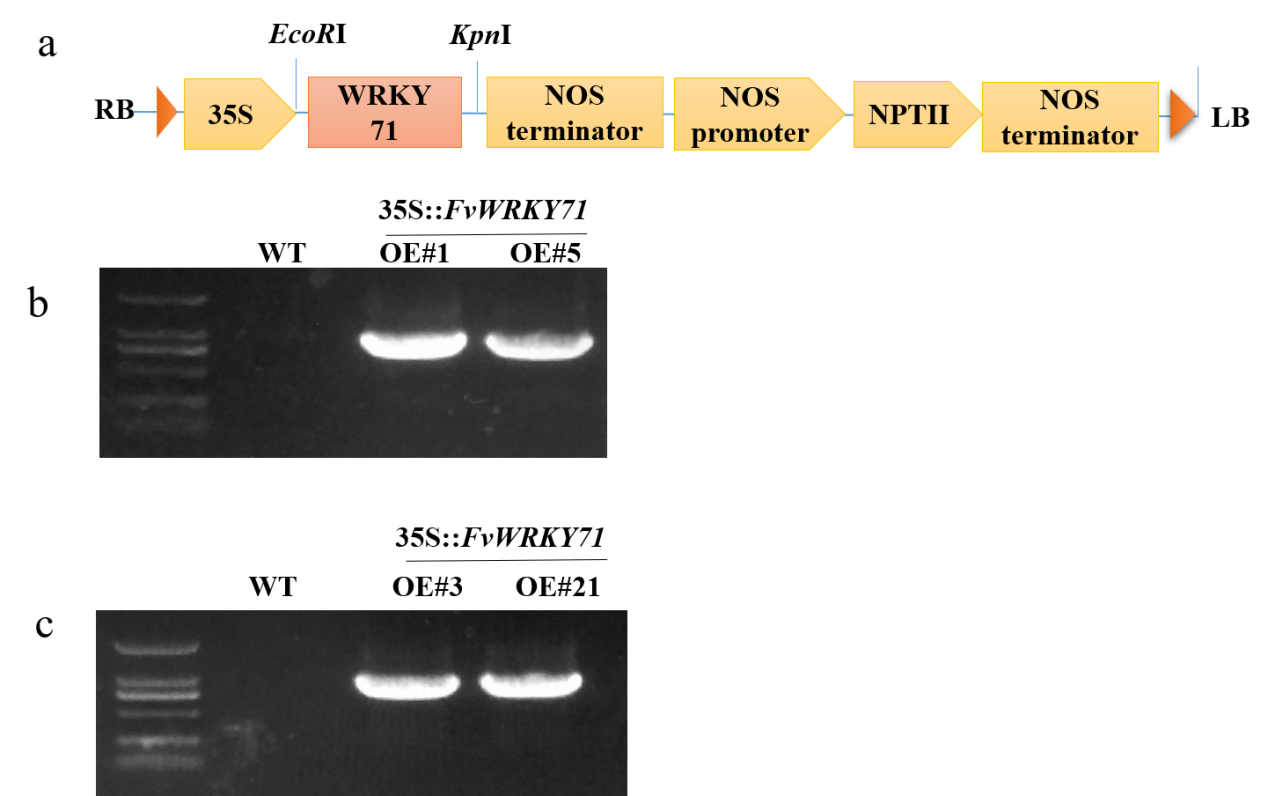


**Supplemental Fig. S2.** Schematic diagrams of the effector and reporter constructs used for the tobacco transient expression assay.


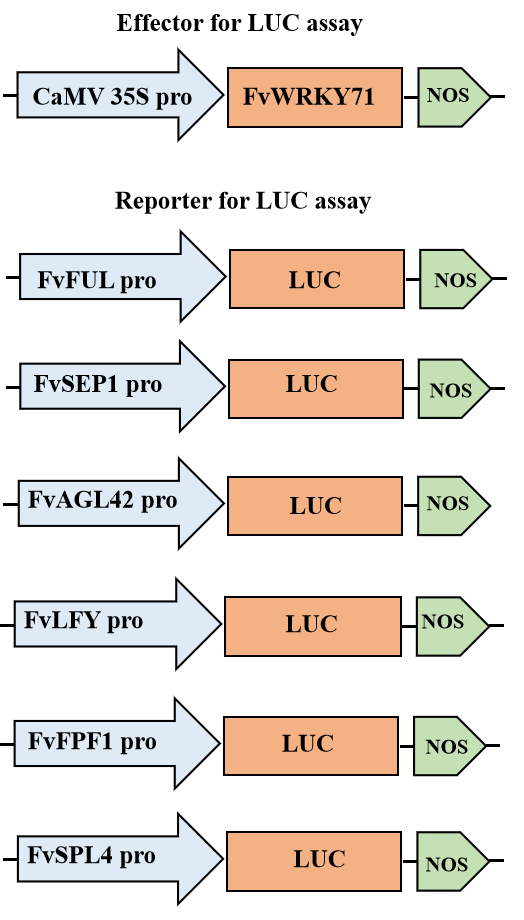


**Supplemental Fig. S3.** The relative expression of *FvTFL1* and *FvSOC1* in *FvWRKY71*-overexpressing transgenic woodland strawberry plants. Values are the mean ± SD from three independent experiments with three biological replicates. Different letters indicate significant differences (P < 0.05, based on Duncan's multiple range test).


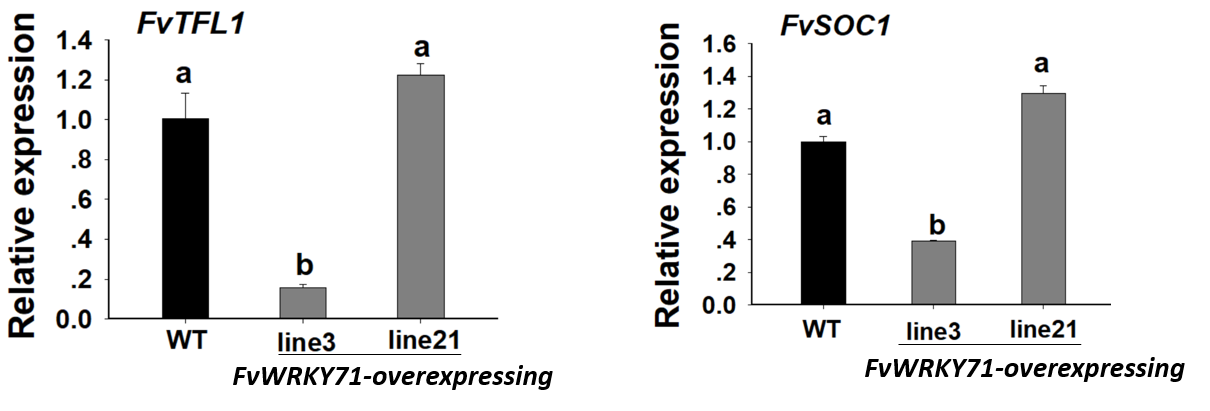


**Supplemental Table S1.** Primer sets used in this study.

| Primer | | Sequence（5ˊ→3ˊ） |
| --- | --- | --- |
| Clone of FvWRKY71 and subcellular localization | | |
| FvWRKY71-F | | GCAggtaccATGTCAAATGAAAAGAAAAGCCC |
| FvWRKY71-R | | GCAgaattcTGGCTCCTCCAGCTTGTGAC |
| RT-qPCR of FvWRKY71 | | |
| FvWRKY71-qrt--F | | GGTGCTGCTCGTCATCAT |
| FvWRKY71-qrt-R | | TTCTCCATCTGTAGCCATCTTC |
| qPCR of Fv26S | | |
| Fv26S-F | | TAACCGCATCAGGTCTCCAA |
| Fv26S-R | | CTCGAGCAGTTCTCCGACAG |
| Transactivation assay | | |
| FvWRKY71-N-F | | GCAGAATTCATGTCAAATGAAAAGAAAAGCCC |
| FvWRKY71-N-R | | GCAGTCGACGACTGCCTTCTGTCCGTACT |
| FvWRKY71-C-F | | GCAGAATTCAAGAATAGCCCTTATCCTAGAA |
| FvWRKY71-C-R | | GCAGTCGACTCATGGCTCCTCCAGCTTGT |
| RT-qPCR for Arabidopsis | | |
| AtAP1-qrt-F | | CAATGAGCCCTAAAGAGCTT |
| AtAP1-qrt-R | | GGGAGGCATATTGTGGCCTT |
| AtFT-qrt-F | | CCCTGCTACAACTGGAACAA |
| AtFT-qrt-R | | TGACAATTGTAGAAAACTGCG |
| AtFUL-qrt-F | | GGAGAAGAAAACGGGTCAGC |
| AtFUL-qrt-R | | ACTCGTTCGTAGTGGTAGGAC |
| AtLFY-qrt-F | | CGAGAGCATTGGTTCAAGCA |
| AtLFY-qrt-R | | TCCTTCATACCCACAAGCGT |
| AtSEP1-qrt-F | | TATGGGAGGAGGAGGAGGAT |
| AtSEP1-qrt-R | | TGCGCCTGAGCTTGGGTTGT |
| AtSEP2-qrt-F | | ATATGATCGGCGTGAGACAT |
| AtSEP2-qrt-R | | TTGGGACTGACCTTGCACCG |
| AtSEP3-qrt-F | | CTCTAAGACTAAGGTTAGCTG |
| AtSEP3-qrt-R | | ATTCACACTTGGTCCTGCTC |
| AtFPF1-qrt-F | | AACCCTAACCAGTCAGGATC |
| AtFPF1-qrt-R | | TGGTGAAATCTCTAGGCAGG |
| AtAGL42-qrt-F | | CCAGCAATCACGACTCACAA |
| AtAGL42-qrt-R | | AGCCTTTCTTTCTCGGACCT |
| AtSPL3-qrt-F | | GAGCAAAGCCAAACAGTACCA |
| AtSPL3-qrt-R | | TTGTGCTTTTCCGCCTTCTC |
| AtSPL4-qrt-F | | TCTCAGGACTTAACCAACGCT |
| AtSPL4-qrt-R | | GATTCTGCATCACCACCTGAC |
| At18S-qrt-F | | GAGAAGTTACTCCGCAACCT |
| At18S-qrt-R | | GAATGATGCGTCGCCAGCACAAAGG |
| RT-qPCR analysis of woodland strawberry | | |
| FvFPF1-qrt-F | ACCCCGACCTGTACCAATTC | |
| FvFPF1-qrt-R | ACGTGGAAGAGATTGGGGTT | |
| FvLFY-qrt-F | | CCGGGTAGGGCATTAGGTAT |
| FvLFY-qrt-R | | TCTGGACCTGGGTCAGAAAC |
| FvFUL-qrt-F | | GCAGTGCATGAATCCCTTTC |
| FvFUL-qrt-R | | GCTGGTGATTTTGGAGCTTG |
| FvSPL3-qrt-F | | GTTTCATGCCAAAGCTCCTC |
| FvSPL3-qrt-R | | GTGTCCAGCTAAGCGTCTCC |
| FvSPL4-qrt-F | | ATCTACCATCGCCGCCATAA |
| FvSPL4-qrt-R | | TCATGGAACCTGCTGCATTG |
| FvFT-qrt-F | | TCTCAGGGTGACTTACACTTCT |
| FvFT-qrt-R | | GTTGGCCGTGGACTTTCATA |
| FvSEP1-qrt-F | | TTCTGTGATGCCCAAGTTGC |
| FvSEP1-qrt-R | | CATTGGTGGTAGCGGTTGAG |
| FvSEP2-qrt-F | | GGGAAGAGTGGAGCTGAAGA |
| FvSEP2-qrt-R | | AAGGGTTTTGAGCATGCTGG |
| FvSEP3-qrt-F | | TCAGATCAACACGGACCCAA |
| FvSEP3-qrt-R | | TCGCACTCTAAGGCTTGGAA |
| FvAGL42-qrt-F | | TGACTTGGAGGCTTTTGGGA |
| FvAGL42-qrt-R | | GATCCTCTCCTTCGCTTGGA |
| FvAP1-qrt-F | | AGCTCAGGAGGTTCATGACTG |
| FvAP1-qrt-R | | TAAGGTCGAGCTGGTTCCTC |
| FvSOC1-qrt-F | | ACACCATTCGAGCAAGGAAG |
| FvSOC1-qrt-R | | CGATCTCCTTTCTGGCAGTC |
| FvTFL1-qrt-F | | CATGAGCTCTTCCCTTCTGC |
| FvTFL1-qrt-R | | GATCACTAGGGCCAGGAACA |
| Yeast one-hybrid analysis | | |
| AGL42-F | | AAATGATGAATTGAAAAGCCTGGCTATGAGCTCTTTGAGCT |
| AGL42-R | | AGATCCCCGGGTACCGAGCTCCTCCTATACACACATCACAAC |
| FUL-F | | AAATGATGAATTGAAAAGCCTAACATCTTTGTGTGTGTCTG |
| FUL-R | | AGATCCCCGGGTACCGAGCTCGGATCTCTCTCTTTCTCTCT |
| SEP1-F | | AAATGATGAATTGAAAAGCTTTCCGAGAATCTGATTTTGGC |
| SEP1-R | | AGATCCCCGGGTACCGAGCTCGAGCTCTCGCTCTCATACAG |
| LFY-F | | AAATGATGAATTGAAAAGCTTCCATAATGAATAGTAACACA |
| LFY-R | | AGATCCCCGGGTACCGAGCTCGGCAACGTAAACATGGCAGA |
| FPF1-F | | AAATGATGAATTGAAAAGCTTCGTTCTACGTAGTCTATTCA |
| FPF1-R | | AGATCCCCGGGTACCGAGCTCAGTGAGAGAAGTTTGACC |
| SPL4-F | | AAATGATGAATTGAAAAGCTTGACCATGTGAAATTCAGGGT |
| SPL4-R | | AGATCCCCGGGTACCGAGCTCTCTACAGTCTGCTATATATG |
| Luc activity analysis | | |
| AGL42-F | | CGACGGTATCGATAAGCTTGGCTATGAGCTCTTTGAGCT |
| AGL42-R | | CTCTAGAACTAGTGGATCCCTCCTATACACACATCACAAC |
| FUL-F | | CGACGGTATCGATAAGCTTAACATCTTTGTGTGTGTCTG |
| FUL-R | | CTCTAGAACTAGTGGATCCGGATCTCTCTCTTTCTCTCT |
| SEP1-F | | CGACGGTATCGATAAGCTTTCCGAGAATCTGATTTTGGC |
| SEP1-R | | CTCTAGAACTAGTGGATCCGAGCTCTCGCTCTCATACAG |
| LFY-F | | CGACGGTATCGATAAGCTTCCATAATGAATAGTAACACA |
| LFY-R | | CTCTAGAACTAGTGGATCCAACAGGTGGCCGCAGACGAG |
| FPF1-F | | CGACGGTATCGATAAGCTTCGTTCTACGTAGTCTATTCA |
| FPF1-R | | CTCTAGAACTAGTGGATCCAGTGAGAGAAGTTTGACC |
| SPL4-F | | CGACGGTATCGATAAGCTTGACCATGTGAAATTCAGGGT |
| SPL4-R | | CTCTAGAACTAGTGGATCCTCTACAGTCTGCTATATATG |

**Supplemental File S1.** The promoter sequences of *FvAGL42*, *FvFUL, FvSEP1*, *FvSPL4*, *FvLFY*, *FvFPF1* and the distribution of W-boxes (TTGACT/C) in these promoters. The green highlighting indicates the W-box sequence.

**Promoter of *FvAGL42***

GGCTATGAGCTCTTTGAGCTTGGGCTTGATGGACCGCCCATGAATCAGATTCTTTTTTTTTTTTTTTCTTTTTTTTTTTGGAATAGCATGTAATCATAAATTCTTAACGCATCATACTTCACAATCTATTAGAGTATTGAATAATATATATTTCACTATCTATTGACTCCATTCAAGTATTTTGTGATCTTATTGCACCACTAAGGATTGTAAATGGATTCATCACTTTATTTACTAAGTTGTCTTCATCTTATTCACTTTAGCTACATATGTAGATATATAGCCTCACTTTTGTATAGCTCATGATGTTTTGTTCAAGTAGCAGTTAGATAAATAAGGTAAAAGGCTAGGTGAAACATTTTCTATGTTTTTATTGGGGAAGAAGGGAAGGGTCTCATTTGGGACATCATCTACCAGTGTAACTTCATCAATACACCAACTCTAAGTGAAATATTGGCATGGCATTTTAAAATGATCTAAGAAGGGAAATAACAATTTATTCGTTGTCGATCAGATGCTAATAACATAATACTATTTTATGTGATCAATGATAGCAAGCGAGTCTTATTCAATTGTAAGTACCTTCTTTCTTTTTGAGTAAAAAATTTACCGTAACGCGATAAAAACAATCAAACACATAAACTTGTAAGTTTCTTCATCTCAATCTATCTATTGATGAAAAAAATATGTAGTATACCTGTCACTAACATAATGTCTCAACTAAGAAGTAGCATTCACTTAACAAGATATTATAATTTGCAAACAAACACATTTGACCTTGCTACACAGCCGATCGATCCAAAGATACTTTCATATATTGTAACCCAAAAAAAAAAAGATACTTTCATAGTTTCATTTCATATATCGTCTCCAGCAAATGGAAATTGACCTGGTTCTGGTTCTGGTTGGTTCTTCATCCTCGAATTCTCTTTACATATTCTGCCGCCAAGAACGTCTTTCCTAAGTACTGTATTCCCCAGCAAAGACATTAGGTTTTGTATGAATTTAAAACTTAGAAAAAAAAAAAAAATAAAGTACAAATTAACGCGCCACTCAAGGTTAGCCACGTGACACGGTCCCCCATTGGGGAATATGACGAGAGCGTGCTGACCACAGACGGCTCGCTGGCACGCTCCTATTGGCCAGTCAGGACATCATTGTACGATGTAAATTTAAATAAGGGAAAATCGAAAGATAAAGATCTAAAAAAAATCTTCTTCATCTGACTTTTGACTTTTGAGAGAAACATTTCAGCTATTTTCTGTTTTCTTTTAATTCTTTGCCAAAACAAGATAAGCAATGAGAACGAGAGGTTCGAGCATCCCTGAATTGTTTATTTCATCTTCTTGGTTTTTTTTTTTTTGTTGCAATTATTTGTTGCTTTGTGGGGTAAGCAACTTGCGAAGCAGGGAAAGACATGGAGAATAATGGACGAGAGGTATTGATGAAAATGGAAAAAAAGGGTTGTGTGGCCTCAGAATATCGAGGTTGGTACATCATCAACATTTCTAGTATAGGATAAAACGATAAAAAGTCATTCTTGCCTTCACTGCACTGCTCTAGCTAGGGTTTCTGATCTGTTTTTCCATATCTGTGTAACTTGATAGCAGTCCTTTGTTAGCTGAAAAGTTACTGGTTCTTCATCATGTTTCCAGAAACCTAAAGCTGACAACTTTCCTGGGGTTTTGCTTCTGGGTTTTCATCTCTGATCGAGATTCTTTGGGGGTTGAGTGATTAAACCTTTCCTGGGTTCTGACTGTCCAGGTTTTAACTCGATCTTGTTCTTTCTTTCTTTCTTTTTTTTTCCCTGTCTGTATTTCTAATACTGTTGTGTTTATCAATTTTTGAAAGAGTTTAGGGATCTGATGGTGATGTTGTGATGTGTGTATAGGAGG

**Promoter of *FvFUL***

AACATCTTTGTGTGTGTCTGTGTATTGACTTCATTCCATGTCTCTATTTTACAGTGTAAGATCAACACTTTAATCGATTCCTTTAATTAGAGCAATGGAGTTGTTGGTTTTCCGCGCTTTTACCAAAGGCCTGAAGAAATTTGTACATTGCTTCGCTGCATGATCGTTATTCGTTGATTTGTGGATATACTTTGTACACATCAATGGTTAAAGTGTTTTTATGTATAATTGAGCAAGGTGCATGCGTAACTTCGATGTCTATTTGTCAAGAACATCATGATTAATGAGTGACCTCTATGAGTATAGTAAAAAAGTTGTATGAATGAATAAATTAATCCGTTTCTTCCTAGTCAAGTACGGTTATTGAAAACAAGTATACATATTAAAGACTTAAAATCTAGGGACACTATGGCGTCAACCCTAATTAGATGGATACTCGATCACTAGTCAATACTACTCAATAATATACTCAATATATACTACTCAGTCAGTTTAGACTAAGATCCACGAAAGTATCCCATCAAAGCCAGACTAATCTCTTACCGAATCAAAAGACCTCTCAAACTTGCTGGACATAAGCTGGTGGGAATCATGAGACATATAGAGGTTCCATACTAGGCAGCTCGACCAACTTTTTCACACTAGATGGTTTTCCTAAATAAAAATTTTAATTCGTGAATGGATGGAATCAATTCCCTTTTGTTTTTAAAAGAATCCCCTCCTTTGAATCTTTGAGCAAACGATTGGTAAAACTAATACCTAACTCACTTTGTACGTAGATGAATCTCGGATTCCTGATGAGTCGACTCCAACTTATCCTATCTTCATCGAAATTAACTCCACGATAGTAAAATGGCAAGTTCCATATTGTAAGTGCGGTAAAAACATTACTGTCAAGGCTTGAAATTCAAAACAAAAATGAAACCTCTAGCTACCAACAGTGGCCCTAGTTATGTTCAATGCCTTTCTTTTCTGTTCGGACAAATAGAAGAAAGGGTATTGTGTAAACCTTTGCTACCAAAGGTGAGCATTCTCGTTTTTTACTTTGACCGAAGAGAATTGCAAGAAATCGATCATCACATTTCTCATATTTGTACTTGGTGCTATACCTTTTCTTTTCTCTCTTGAAGAAAAAGTATCATTCATATAGGAGTATAGAGAAATATTGGCTAAAAATTGTAGGGTACTTATAAAGAGGAGTGTACATTGGTTCCTGCCGACTGTTTTTGAGCAAACACCACTCATAATTAAGTCTTCCCAAAATGCGTAGAATGACTTGAGCAAGTCAACAAGGAGCCTCAAAGAAATACTGAAACAATCAAAATTGAAGTAGTAGATTTTTGGCAACGCAGACAACACCCTGGTTTAAGATACGTAAAGTGTACATGTTGTAGAGAGAAATGAGGTTCAAAATTTAGTAGATCTTTCGGAACGATAGGCAAAACCCTATTTCGAAATTGTCTACTATGTTGTAGGAAGACACAATAAATTATTGGTATTGTACATACGATATCAAAGATTATTATTTGTATCACAAAAGAGGCTAAATTCCAATTAATTATTTGTTGCAAGTAGAAAGGCATGCCTCATGACTAAATCAAGAAAGGGTACAAAATACAATCTAATGATATCTCACTTTCATTAAAGCAAAAATTGGATTGGCAAGCATATTACTCCTTTGAGAACCCTGACACAAATTAACGAACACTATAGTGCCCACAGCATGATACTCCACAACAATGCTAAAATATATTTGGCCAAGAACACACATATATAGTAAAAATCCACAGTAACATCATGAAAGGAGCATCCAAAGAAAAGTAAAAAAAAAAAAAAAGAGTAAGAAAAAAAATTACAGAAGAAATCATAGAAAGATCCTGTGTTTCAGAAACCCGAGCTCGTACGATGATGGCGTCGTTGGTAATTAATTAAGCTGAAATAAGGAAAATATGTGGTTTCGAGGCCATGTCTTATAGACCAATAAACGACTGTCACAGAAGCTCCAGCCAATAGCAACTTGACAACGCCTTGGGAAAAACCATCCGGTTTTGGAAACCCGCTCATCTAATCCCACAGAGGTTTTCAACTTTCCTATAAATTTACATATAATAAAACCACAGATCAGTCACTCCCAGACTAGCAGACCATCTATTTAGATATATAGAAAAAACCCCATCTATATTCTCCAGCTCTTTCTCTCTCTCTCTCACTCTCAAATTCTTTCCCATTTTCTTGGTGTTTTTTTTTTCGGTTTCCCAGAGAGAAAGAGAGAGATCCATTATC

**Promoter of *FvSEP1***

GAGCTCTCGCTCTCATACAGTCAACTTGAATTCGGTGGTAGTTGCACAGTGTTGGTGGCCTTATAATTTATTTGGTGAGGGGCACGTGTCGGCCGGAGTACAACGGCCATGTCCACTCTCCAACTTTAAGCTTGAACAGTGACGAGAGTGGGGTTGTTTGCTGAAGGAAACAGAATCAGATGCGACTCAAGCAGGGAAAGGGAACAGGTTTCGTATCGTTTGCTGTTGAATGAGAGAGTTTGAATGCCAGCAAGCGAGTAAGGTCTCGACACGTATCCGGGCTCCAAGGGGATTGACTGCACTGGATTGCTTTGTGTTTCGTTCAGGCCGTTCCTGCGGGGAGGTGTGAGAGTCCGCCGTCTCAAACACCACAAATGCGAGGACACTAAAAAAATAATATATATACATATATATAATTTTCATAAAACTTTTAAATTTTTTCTTATTTCTCGTTTTCTCGCATTTATTATATCTGACTAATATTATAATACTTTATAAATTCATATTTAAGATTCAAGATTGTACATTTACTGTGTTTTTACGTATCTTGTGGAGAAATTATTTTTTTGCAACGACCATATTTTTTTTCTTTCATATGACCGTTAAAGAGCACAATTCTCAAGATTCACCTCAGGTCTTCAAATCCCAGGTCCGGCCCTGGTTTCATTTCTTTTCTCAAGAACCAATAGGCATTTTAGTATGCATTTTAGTCGTTTGGAAATGCATGCTAGAATGACACATTACAGTGTCCATATTATGGGGAAATCGTTTCACTGCATTTAGACAATTTTGATTACTTATTTGAGCCTATAAACTCTCGACATGTTTCAAACACATAGAAAACTATATAGATAGGCTCGTTAATGCACTCAATTGTGGTGTTCCAGAACTATGATCATCAAAGTGCCAAATAATTTTAAAGATCATAAGACACGTACATATGCTTGGATTTCTAATAGACGCTCCGAAACTCACACCACCTTTGTACGGAACATCGACATCAATGCCTAGAGATGACACTCAAAACTTGTCACCAATCAACCTTCCAAAACCACACACACCTTCTCTCTTGACCTCATCATTGTGCAATTAAGTACCCGTTCGGCAACCAACAAAATGACTTGCCATGCCAACGGTATCATTGATCATTTTTGAACATGGCACCATTTTGGTGGTGTAGTTGCTCTCTCTCGGCGCTAGGGCTAAAAGAGAGTTATGATGAAGGATAAAGTGTAGAGACAAAGAGATAGCAAGAGAATCATCATCTTATTCGTTGATAGGAGCCTTTTATATAGGGAATTACACAATACCAATATGGTAAGGATATGAATACATAGATCTAGTCTAACTACATATCCTATTGGCATAAGGCCAAGGCACACATAGAGAATATCCTAGAACACTCCCCCTTATGCCGCGCGCTGATATGCCGATGGTGCTGATCTGTTGCCTCGTCAAAAACCTCGTCATGTCACAAAAACCCTGTGGGAGAAAAACTGAACCTTGATCGTAGGAGAAAAAGAGTACAACGCACCCTTCACATTTGATAGTGACATGTATGTATGTGCTAGACTCCCCCTGAAGTTCACACCTCCCTCTGATCTTTACATCAATCATAGGGCTCTTCCTGATTCTTACTAATCACGGAAGTTTCAAAAACTTAGCATGTCAATGCTCTTTAACATGTCTTCAAATGTGGCATTAGACAATGATTAACTAAATCATGCCGTATTGTCCTCAAATGATCTTTTACACTTAGATCTTGAGGAGAAGGTCGCTTGTGAACTCAAAACATAAGTTCGTGCATGAAATCAGGTTTCCGCGCAGCATGACCTTCAATTACTAGAACAGTCGTAACTTCCTCTAGGAAATACATATGAACGAACCGTAAACGTTTTTGGAAACTAGACTCATTTAGCTTTCCAACCGTATATTAGACACATTCTGGTTCATCAGGAGCTGTTCACAAAATCCCGTCGAAGTTGACTGTTTTGCCAAAATCAGATTCTCGGA

**Promoter of *FvSPL4***

CAGATTGCTGAACTTCAGAAACTAAGCTCAAGCTCCAAACCGTTAATTGAATAGTGAAAACATGATGAAATATAAACATCTGCTTCCAAACCAACTACGTACCCTGAACATTTGTGAAATAATTAACTTATATACATCCTCACTCTTCTGAAAGATATAATCACTAACCAAATAACATGATGAAAGCTTTAGAAATATGTAATTTTAGTGGCAGCAAGCAGACATAAAACTCTATCTCGATGTTGACATGCAAAGGTACCCACAATGCAAATCTGGCTTATTGCATACCAGTTGGGAAGAAGAATTAGTTGCTATCTTCTTATTCTTTCACTTCCTTTTAAGACGAAATAAAACTTCGAGATCAACTAGATTAAAGTTGAGGTCCACAAGGTACAGAAAACCCCATGAAATGCATCAACCATTGGGTATCTGAGTACTATGACCATGTGAAATTCAGGGTCCTATCGCATTAAGAGGTCTGCCTATATAGGTACCCTTTTCTGATACACAACACATCCAACACAATAAGGTCCCGTACGTGGGTCAGCTAGCACCATTACGTATATACGCCTTGAGCTATTGCAAATCTACACAGTCTGTATATTAAGTTAACGCATCCCTAACGTACCCGTATAGTAAAAATCCATATACGTACTGAGGTTAGAAACTTGGGCAAGGGTCTTGTTGTGTTAGTATAGTTCTCATTTACAGTAAATGATGCTGTAATTCTAATGCAATATCAGAGCAAGTATTGATATGACAATTGACACATGACTTGAGTTCTTGACTTTAACACCGGAGGCGGAGCGTGGAGTGGTTCCAGCCCTAGAGCTTTCCCAGCGGGAAGTGCTTAGCATAATACAACACCATGGTGGTTTTGAAGTGTATGCTCTAATTTTAAATTGGTAAATGAGCAGGAGTGTGCTGCGAAGATGAACTGGATATCTTTCAATCCTTAGCCAATCGACATCTCTCTATCCTTAATTAGACAACTGATTGGGGTTTGTTGCTCTAGTTCTAAGTTCTAACAATCATCATGGATCTATTTTAAGATGATTAACTGTGATCATGTAATTATATGTGCATATATAGCAGACTGTAGATTCTACATACAATAAAGACATGGGAGTAAAGTAGATGATCGAAGATATATAAGAGAGAAGGCATTCACCGTAGATTAAAGACCTAAACCTTCGTCTATGGCCAGTTTGATTGAATAGAGCAACAGAAGTAGTCAGTTGTACTGCATGTTAATTTACTCCGATCCAGTACGCAAATTTAATTGCAGCATGCATATCTGCTCAGCTCTGCGCCTTTCTTGTTCACTTGGAACCCGTCATGTCTCTTTCAATTAATAAAGCTTATTACGATCTGAACTACTACGTACTACGTACTCTACAGGCTTGTGATATATGTATTTACACACTTGCTACTCAAGATTTCAACTCGTCTAATAATCAATTAGACTCGTCTAATTAATCAATTAGAGAAATTATGCACAAATAATTTCTGTGTGAAAACAAAATCATGGAAACTAACCAAACTGATCATATTTTGACATGTATAGAATATTGTTGTGTTTTCAAAGTTAGAGACTGACAATCTTCGGCCTCTTTTCCTCATAACAATATTGTTCTTCCTTAACCACCTTTAAAGCTTGATATTTTATCTAAAGAACCTTAATTTAGGGGTGTTTAAAGAGTTTTGCATAATATAAATTAGGGTTTAGGACAGGTTGTTTGATCCGCAATTCCCAAAACGACAATGTAAGACCTGGACAGGTTATTTGATCCACATGATAAGAGATGAGTGATATAAGTTAATTATGTCGAACCAAAGAACCCTCCTCCATAAAAGAAGAGCTTTATACGATGTTTGAGACAAATTAATTCGTCGTCTTTCAGTTTGAACTATCTTTGTTATGTGTATTCACCGTTCCATTCCAGTTTTCACCACCTTAAGATATTGACACACTCGGGCTGTCACAGAAAATGGAGTTGGTGTAGTTGTGGTCCTCTTCCACCTTTTTTCACCCTATTTATTCAATACACCTTCTTCAGGACCTTCATAACTCCAAGTACTTCCTAACATACTGAGTGAGAGCTAGAGAGCTATCTTAGCTTTCATCA

**Promoter of *FvLFY***

ATTTAATTTAGCAATACTGCTGCAAATACTTTTAGCTTGATTATAACTTGACGTATACTTAATTTATCTTCTTATATATATTTTGTCGACATATCAATTTGCATAGTAATGAATCAACTATAGGGTACCCCCTCAACTATGTTCATCATTTAGTATGTGCGGCCATTTGTAATATATCTTCGCAATAACCTATACATGACTATATCTTTGTTATTCGACCATGTGGAACCAAATTACCTCACTCGACCACTTCACCACATGGAAGATCGATTCAATTCAATCATCATCTGCCTGACTACCTCGCAGGGGTTCATCTTACGTGAACTACTCCAAGTCAGATTGCCATAGAATCATAGATAACCATAATGAATAGTAACACAAGCACACATTGCTCCCCCAACTAATTCTGCTCCCAAGTAACCACCACGTACTATGCCCATCACTGTGCCCATAGTAAAAAGTACACTTTATAGGTGAGAGAGAAGTAATTCCCAGGTCGAGAAAGCACTGAAACGACAAGGTAAAAGGAAGAGCATAGCTCAAAGTAAAAGTGTAAAACCATTCAACGTAAAAGCCAGTGCTCCAGAGTCCAGACTCTGCCCTGAGCCCCGACGAGTCATCCCCAACCCAGAACAAGGTACACGACGTGTCGACCACCCATTGGTCCCTACCTCAAAAAAAACCGTCTTGTTGACCAAATATTAATTAATAATTTCCATCCTCGTCTCAAAAGAAGAGAAAGTCGAAGCTCCAAAACAAAGTGCGCCAAATTCCAAATCTTCCTGCACCTCCCCCATCACGTCCCATCATTCATGGCCCACCACCCGATCCCCATCTCCTCTCCTCCGCCCGCCGCGTGGTGCTCGTCTGCGGCCACCTGTTGGATCCCCGGACGGGAACATGTATGCCGCCGCTACAGGCGATCGCTGCCACCCGGCGATATCTCAGTTTTCATCTTTGCAAGTACAGAAAGAGAAACAGCCTTTCTGATCGAGACGACTGACCCTAGTACCCAGGCTCTCTGCCATGTTTACGTTGCCAAGGAACAGTAACTATGTATTTACTCTCCCTCATGAAACTACGAAAAGGCCCTTGCATGCTTGTCGTCTTCGGGTAGAGTTTCAGATTTGAGATTCAGAAAATCTTACACAAGATATGGGCTCTCTCCAGGTCTCAGTGTCCTTTGTCCTTGAAAAGCAACAGGGTTCATTATGATGGATAGTGATCGACGGAAGGACGAAAATTACCATCTATTACCCCAACACCAATCTCAGTCCTGGAATTCCATTATGCCAATACTTGCAAATATTGCAAACTTTCGGGGAGGTCCGAACTCCGAAGGGCAAGTCTACTGGGAGTCTTGTTTTGCCCAGTAATGTTGACATTGTGTTCTTAGCCCTAGCTAGGTAGTCAATTGTTTTACTAAATTAAATTGGGGTTTTACTTGCTACAGTAAAAAAAAATGTAGGTTTGAGTGATTTCAACCAATCAAAATTGCTGTTCGATCACGTTGTTAATAATACTGCATGCAAAACCTAGGGATTTAGAAGTGCGTGGAAATCTGGCAAGAAAGTTAGCAATTTCAGAGAATTGCACAGGCTTTGGGGTTAGACATTAAAAAACTAAGTAAAACTCGTAGGTAAAAAAAAATAAATTGAAATTTACTTATCGAGCGGGATCACATTCTGCAATAACTTGGTTTGGTTTGGATCCATCCCCAGTTTGGGTATAGTTCCCTCCTCGGGAACAATCAAATGCAGGGGTAGTTTGGGGAAAAAAGGTCAAATAGTTGTCTTTATAGGCCGGAACAGCTACAGCTCTCCCCAACCACAAAATAGGTGAGTGTTCAGCTACGACTAAACACTGACACAGTGAGAGATAGAACAACACTAAACAGAGTTAGGGTGAGCTGAGCTGAGCTTC

**Promoter of *FvFPF1***

AAAAAAACAAGAAAATTAAAACGTTCTACGTAGTCTATTCAATTCGTTGTCCAAGTTATATCACCTCATTGAGAAGAAATTTGGAGGTTGACTACTCATGAATATTCGTAACAAAGTTTTCCTTTTCTTTCTTTTTTCTTCCTAACAATTAATCATTATAGATTTATAGCTAATAGTTACAAATGGTGCGCATGTATCCCATATGTGTAAGCATGTGATTGTTGGGGCTACTTGGGAGTAGTTTCTCCAACCGGAAAATATCTTAAGGCACAACACTAATCATGGATTTGACAAGACAACGCTCAAGAACACTCTCGACGTAATTTGAGGACCATCTAGCCCATGTGTGATCACATGTGGGCATCATGGTACATTACACAACCAATCTCATATTTTGAATGAGGGATTGTGGGAATTGTGTAACTTGGTCACGAGGTGGTCCTTTTGATGACAATTAGGGCACGTAACATTTAGGGTTCAATTATGATCGATTTGATCGCGACGACAGTGTTTCATCGATTAGATTTGAGTGGTGTTCAGAACTTGTGGAAAAGACCAATGATTATAAAATGATTATGTGTAATAATTGCCTGTCTAATCTCGATCATAAAAAGAACTCGGTAATCTACTGTTCATTATAGTGATCCAACTATTACAACCTCGCTTGATCCAAATTTAGTCTTTAAGGAATGTATAATTGTTTGGTCAACATGCTTGATACAGTTTTCATGGTCAATCTTGTTATCCAATTTAAGATCTTTCTGTTTCTTGTAAGGAGTAAGTACACTATAGGGCAAGTAAGTTAGGAGAGTGCTTAATTCCGGTCCGGTTTCTTTCCTGGTATGTTTAGGAAATGTCATAGGGTTTCCTTTGATTTGGTTTTTATCAAGTTTTGTTGCATGCTAGGTAGCTTTTTTATAGTTCTAATTGGCTTTAGATCTTTATGACTTCCTTCGGATCATAGCCTTTCGAGCTGAGTTATGCTTATGTGACATTTGCTATATATGTTTTAATGGATTGACCCCCAATTTCTCTATATATAAAAATTAAAAACAATTTGTAACAACTCTCAAATTAAAGAATGTACTTAATCCGTTAAGTTAAGATAGGGGTATAATCACTGCTTAAGTAAACAAGGCAATAACTTCTGCAGTCCTAGTCTAATGTCGTAACACACAACTTGAGCAAAGCATATATAGACGTCACACAACTATATATAAATATCCACAACATATATAGCAGATGGATCAATAATGGGGACAATGCAACGTGAGTAAAGTGAAGTTACAGCTCTATTAAGGTTTCCAGATTGCTCTAGTACGTAGAGCAGAAGCATCAAGAAGACTAGACTAGCCAGAAAAACTGAAAAGAGAGAAAGCCCTAAACCCCGAAATATCTAAACGTAGTAGAAACGTGTACCCAGAACGATCTCCATCTCCCACTCCCTTCACGTGGACATCTCGTGATCGATCTCTGTGGCTAACATCATTGACTACAAATTGTTTAATGAACAGTGCCACAATAGTGCGCTCAGGGTTACACAGATTCCGTCCCCATTAATTTCGAAAAGCACAGAGAAAAGATTCTCCTCCTCAAAAACCCTAAGCCCCACCGGCAGCCGGGAATCGTTTCCTTGCGATGAGCCCTAGGGTTTGTTACAATGCACCGTCTTCCCCACACTGGCTCCCACAATATCTTGATTCTCTTCAAAAGCCAAACACACCCACACGCCGTCGTTTTCAGGAGACTTGTCTAACTCCCACTCAAACTCCCAGAGTATTTTCCTCTTGTTTTTATTTGTCCCCAACGCTCTTTCATCGCCCTATAAATACCTCCCTCCCCCTTCTCATTTTCGCCACCATCAACAAAACACACACAGAGCTCTCCCCCTCTTTGCTCTTCTCTCACAAAACTCAGCAATAATTCGTGTAGGTCAAACTTCTCTCACT
